# Worldwide absence of canonical benzimidazole resistance-associated mutations within β-tubulin genes from *Ascaris*

**DOI:** 10.1186/s13071-024-06306-5

**Published:** 2024-05-16

**Authors:** Ben P. Jones, Kezia Kozel, Allen Jethro I. Alonte, Kennesa Klariz R. Llanes, Alexandra Juhász, Umer Chaudhry, Sara Roose, Peter Geldhof, Vicente Y. Belizario, Peter Nejsum, J. Russell Stothard, E. James LaCourse, Arnoud H. M. van Vliet, Vachel Gay V. Paller, Martha Betson

**Affiliations:** 1 Department of Comparative Biomedical Sciences, School of Veterinary Medicine, University of Surrey, Guildford, GU2 7AL UK; 2 Institute of Biological Sciences, University of the Philippines Los Baños, Laguna, Philippines; 3 Department of Tropical Disease Biology, Liverpool School of Tropical Medicine, Liverpool, L3 5QA UK; 4 Institute of Medical Microbiology, Semmelweis University, Budapest, Hungary; 5 School of Veterinary Medicine, St. George’s University, True Blue, West Indies, Grenada; 6 Department of Translational Physiology, Infectiology and Public Health, Ghent University, Merelbeke, Belgium; 7 Department of Parasitology, College of Public Health, University of the Philippines Manila, Manila, Philippines; 8 Department of Clinical Medicine, Aarhus University, Aarhus, Denmark

**Keywords:** *Ascaris*, Benzimidazole, Drug-resistance, β-Tubulin

## Abstract

**Background:**

The giant roundworm *Ascaris* is an intestinal nematode, causing ascariasis by infecting humans and pigs worldwide. Recent estimates suggest that *Ascaris* infects over half a billion people, with chronic infections leading to reduced growth and cognitive ability. Ascariasis affects innumerable pigs worldwide and is known to reduce production yields via decreased growth and condemnation of livers. The predominant anthelminthic drugs used to treat ascariasis are the benzimidazoles. Benzimidazoles interact with β-tubulins and block their function, and several benzimidazole resistance-associated mutations have been described in the β-tubulins of ruminant nematodes. Recent research on ascarids has shown that these canonical benzimidazole resistance-associated mutations are likely not present in the β-tubulins of *Ascaris*, *Ascaridia* or *Parascaris*, even in phenotypically resistant populations.

**Methods:**

To further determine the putative absence of key β-tubulin polymorphisms, we screened two β-tubulin isotypes of *Ascaris*, highly expressed in adult worms. Using adult and egg samples of *Ascaris* obtained from pigs and humans worldwide, we performed deep amplicon sequencing to look for canonical resistance-associated mutations in *Ascaris* β-tubulins. Subsequently, we examined these data in closer detail to study the population dynamics of *Ascaris* and genetic diversity within the two isotypes and tested whether genotypes appeared to partition across human and pig hosts.

**Results:**

In the 187 isolates, 69 genotypes were found, made up of eight haplotypes of β-tubulin isotype A and 20 haplotypes of isotype B. Single nucleotide polymorphisms were seen at 14 and 37 positions for β-tubulin isotype A and isotype B, respectively. No evidence of any canonical benzimidazole resistance-associated mutations was found in either human- or pig-derived *Ascaris* isolates. There was, however, a difference in the genetic diversity of each isotype and distribution of β-tubulin genotypes between human- and pig-derived *Ascaris*. Statistical tests of population differentiation show significant differences (*p* < 0.001) between pig- and human-derived worms; however, more diversity was seen between worms from different populations than worms from different hosts.

**Conclusions:**

Our work suggests an absence of canonical β-tubulin mutations within *Ascaris*, but alternative modes of anthelminthic resistance may emerge necessitating continued genetic scrutiny alongside monitoring of drug efficacy.

**Graphical Abstract:**

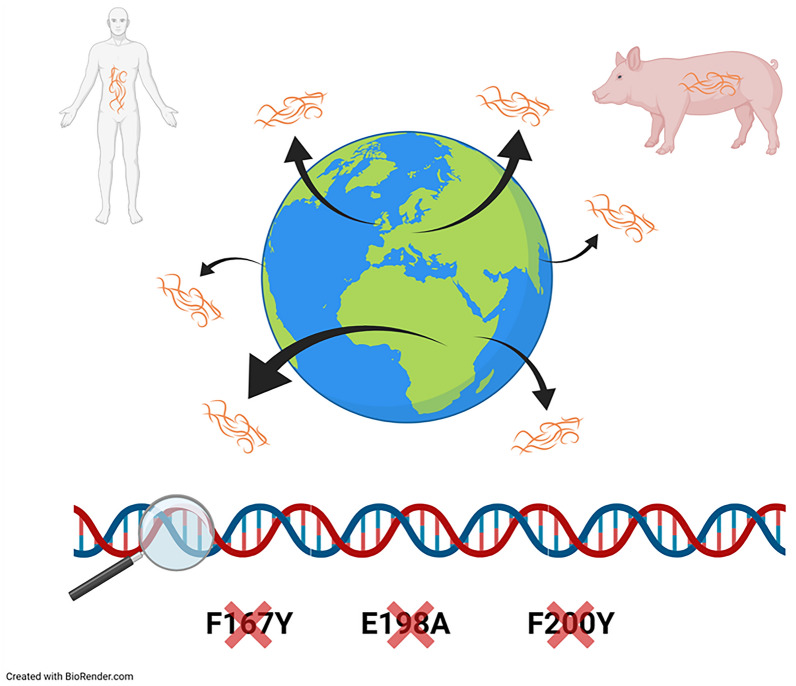

**Supplementary Information:**

The online version contains supplementary material available at 10.1186/s13071-024-06306-5.

## Background

Over half a billion people are infected with the giant roundworm *Ascaris*, predominantly in low-middle-income countries [1, 2]. The WHO 2030 roadmap for action against neglected tropical diseases aims to eliminate *Ascaris* and other soil-transmitted helminths as a public health problem from 96 endemic countries by 2030 [3].

This will require several interventions including improved detection, and the uptake of a ‘One Health’ approach, in which hygiene and sanitation and environmental and animal reservoirs of disease are considered [4]. The incorporation of knowledge from mathematical models and the intensification of mass drug administration (MDA) in endemic regions are also key to reaching these targets [4, 5]. This increase in drug usage, in particular the increased use of benzimidazoles (BZ), could lead to the development of drug resistance, which is becoming widespread in many nematodes of veterinary importance [6–11]. Porcine ascariasis, mainly associated with *Ascaris suum* infection, is a global problem and can have economic effects by reducing meat yield [12, 13]. Benzimidazole drugs are also commonly used in pig production to control ascariasis. There are a few reports of potential BZ resistance in human *Ascaris* populations but none in pigs [14, 15]. Implementing surveillance is therefore paramount to prevent BZ resistance in *Ascaris* from becoming a problem for both human and animal health.

Most studies on BZ resistance in nematodes have focused on single nucleotide polymorphisms (SNPs) in the β-tubulin isotype-1 gene, which have been associated with phenotypic resistance in ruminant nematodes [16–18]. It is also possible for SNPs in other β-tubulin isotype genes to lead to resistance, as has been demonstrated for *Haemonchus contortus* [19]. Most work on *Ascaris* has focused on a single isotype; however, recent work has analysed multiple *Ascaris* isotypes to search for evidence of resistance, with none found [20, 21]. Two isotypes have been studied for *Parascaris*, with significant changes in the expression of isotype 1 in response to BZs observed but no evidence of resistance-associated SNPs [22–24]. There have so far only been a handful of phenotypically resistant isolates of ascarids identified that can be studied, with only two investigations screening for the common resistance-associated SNPs in resistant isolates [25–27]. Analysis of seven β-tubulin isotypes in BZ-resistant populations of *Parascaris univalens* found no evidence of resistance-associated SNPs in any isotype [26, 27].

The three common amino acid substitutions associated with resistance (F167Y, E198A and F200Y) are found close together in the β-tubulin protein; however, in the β-tubulin genes an intron separates the F167 codon from the E198 and F200 codons. Previous attempts to identify SNPs have therefore relied upon separate assays to capture the genetic material at both sites [14, 28–30]. Without the knowledge of which SNPs are most associated with resistance in *Ascaris*, it is vital that all three are analysed to ensure that no causative mutations are missed. The most efficient way to do this is to cover all three sites in one assay. Deep amplicon sequencing is more sensitive than traditional sequencing methods for the identification of alleles at low frequency. This methodology combines the high read depth of next-generation sequencing with the specificity of single marker sequencing and allows high sample throughput. Recent advances allow all three of these SNPs to be captured in a single assay and isolates can be screened for all three simultaneously [20, 31, 32]. In addition to enabling screening of all three SNPs at once, these newer sequencing methods allow researchers to perform population genetic analysis. The outcomes of such work can inform on the origins and spread of resistance and the diversity within and between populations [20, 33].

There has been much debate over the relationship between *Ascaris lumbricoides* (mainly infecting humans) and *A. suum* (mainly infecting pigs) with hybridization being possible and some suggesting that they may be separate lineages of a single species [34–37]. In industrialised countries where *A. lumbricoides* infection in humans is not endemic, *Ascaris* infections can often be attributed to *A. suum* and infection via contact with pigs [38–44]. Recent work in a region of Kenya that does not commonly practice pig farming found that most worms contained pig-associated mitochondrial haplotypes while showing signs of a mixed human/pig nuclear genome [45]. This supported the hypothesis that in the past there has been hybridization of *A. suum* and *A. lumbricoides* that gave rise to fertile offspring capable of being transmitted through humans. This study also calculated that there was not enough divergence between the mitochondrial *cox1* gene to designate the pig- and human-associated clades as separate species [45]. The zoonotic capabilities of *Ascaris* mean that a One Health approach needs to be implemented to control these parasites. Determining the origin of infections will be a key aspect to monitor to prevent the spillback of infections.

In this paper, we build upon previous work to screen global *Ascaris* samples from both humans and pigs for the presence of canonical resistance-associated SNPs in the two most highly expressed β-tubulin isotypes (isotype A and isotype B). In addition, we assess the inter- and intra-farm diversity of *A. suum* isolates from UK pigs and analyse samples from a variety of locations worldwide from both humans and pigs to compare the diversity of these two isotypes between both region and host.

## Methods

### Egg isolation and larval culture

*Ascaris suum* eggs were isolated from faecal samples collected from four UK pig farms. For three of the farms, all eggs were isolated from a single faecal sample as these were the only faecal samples containing *Ascaris* eggs from that farm. For one farm (Farm B), eggs were collected from multiple faecal samples from a pen housing multiple piglets.

To isolate eggs, a previously described salt-sugar-flotation method was adapted with the sugar flotation step replaced by further salt flotation steps and the volumes scaled up to 25 g of faeces and 225 ml of saturated salt solution [46]. The pellet of eggs obtained using this method was suspended in 1000 μl PBS and stored at 5 °C until further use. The thick proteinaceous coat of *Ascaris* eggs can sometimes hinder nucleic acid extractions and the eggs were therefore de-coated. Egg suspensions were transferred to 50-ml Falcon tubes and incubated in 20 volumes of 3% sodium hypochlorite at 30 °C for 90 min in an incubated shaker at 100 rpm. Eggs were then washed three times by centrifugation at 1200 xg in PBS [47–49]. To acquire larval stages, *A. suum* decorticated eggs were incubated in 5 ml 0.1N (0.05 M) sulphuric acid at 28 °C for at least 21 days in 50-ml Falcon tubes. Samples were aerated by hand weekly by opening the lid and gently mixing tubes [50].

### DNA isolation, preparation and sequencing

Genomic DNA was isolated from *Ascaris* via several methods. For larval DNA, pools of two to four eggs containing developed larvae from four populations across the UK were placed in 30 μl lysis buffer made up of a 20:1 ratio of DirectPCR Lysis reagent (Cell) (Viagen Biotech) and Proteinase K (New England Biolabs). For adult worms from Hungary and the Philippines, DNA was extracted from a 2 cm portion of the anterior end of the worm using the Qiagen DNeasy Blood and Tissue kit (Qiagen) following the manufacturer’s instructions. For all other samples, the DNA was supplied already purified and extraction has been previously described [40]. Samples came from nine countries with 151 and 39 originating from pigs and humans respectively (Table 1).

**Table 1:** Characteristics of *Ascaris* samples included in this study

Country	Region	Sample type	Host	Sample no.	References
Bangladesh	Unknown	Adult	Human	11	(Betson et al. 2014)
Belgium	Flanders	Adult	Pig	109	(Roose et al. 2021)
Denmark	Unknown	Adult	Pig	7	(Betson et al. 2014)
Ethiopia	Jimma	Adult	Human	29	(Roose et al. 2021)
Guatemala	Santa Rosa	Adult	Pig	6	(Anderson et al. 1993)
Guatemala	Santa Rosa	Adult	Human	4	(Anderson et al. 1993)
Hungary	Forráskút	Adult	Pig	1	This study
Hungary	Eger	Adult	Pig	12	This study
Nepal	Unknown	Adult	Human	3	(Nejsum et al. 2005b)
Philippines	Los Baños	Adult	Pig	3	(Anderson and Jaenike, 1997)
Philippines	Trento	Adult	Pig	2	This study
Philippines	Buwan	Adult	Pig	2	This study
Tanzania	Unguja	Adult	Human	8	(Betson et al. 2011)
Tanzania	Unknown	Adult	Pig	8	(Betson et al. 2014)
Tanzania	Pemba island	Adult	Human	77	(Roose et al. 2021)
Uganda	Kampala	Adult	Pig	4	(Betson et al. 2014)
Uganda	Kabale	Adult	Human	8	(Olsen et al. 2009)
Uganda	Kabale	Adult	Pig	4	(Nissen et al. 2011)
UK	Devon	Larvae	Pig	20	This study
UK	Clwyd	Larvae	Pig	20	This study
UK	Lincolnshire	Larvae	Pig	19	This study
UK	Derbyshire	Larvae	Pig	20	This study
UK	Cornwall	Adult	Human	5	(Bendall et al. 2011; Betson et al. 2014)
UK	Bedfordshire	Adult	Pig	23	(Betson et al. 2014)

A first round of PCR was performed using BtA and BtB primers (BtA-For, BtA-Rev, BtB-For and BtB-Rev1) with added adapter sequences which amplify β-tubulin isotype A and B respectively, as previously described [20]. Four forward and four reverse primers were used, with each containing the specific primer and an Illumina adapter with between 0 and 3 random nucleotides separating them [20]. A primer mastermix was made for forward and reverse primers containing all four adapter primers at a final concentration of 10 μM. First round PCRs were carried out in 25-μl reactions containing 1 × KAPA HiFi Fidelity Buffer (KAPA Biosystems), 0.5U KAPA HiFi DNA Polymerase, 0.3 μM forward and reverse adapter primer mastermix, 0.75 mM KAPA dNTP Mix and 1 μl template DNA. Reactions were performed under the following conditions: initial denaturation at 95 °C for 3 min followed by 35 cycles of denaturation at 98 °C for 20 s, annealing at 61 °C for 15 s and extension at 72 °C for 30 s, and a final extension of 72 °C for 2 min. Equal volumes (20 μl) of the two separate PCR products, BtA and BtB, from the same samples were pooled prior to purification. PCR products were purified using NucleoMag NGS Clean-up and Size Select kit (Macherey-Nagel) following the manufacturer’s instructions.

A second round of PCR was performed to add Illumina Nextera XT P5/7 indices to each sample [32]. Sixteen forward and 24 reverse indexing primers were used to give 192 unique barcode combinations over two 96-well plates [32]. The second round of PCRs were carried out in 25 μl and contained 1 × KAPA HiFi Fidelity Buffer, 0.5U KAPA HiFi DNA Polymerase, 0.5 μM of each primer, 0.3 mM KAPA dNTP Mix and 2 μl of the purified product from the first round of PCR. Reactions were performed under the following conditions: initial denaturation at 98 °C for 45 s followed by seven cycles of denaturation at 98 °C for 20 s, annealing at 63 °C for 20 s and extension at 72 °C for 2 min.

Twenty μl from each well of both plates was pooled into a new plate (e.g. A1 of plate 1 and A1 of plate 2 into A1 of the pooled plate). Pooled products were again purified using NucleoMag NGS Clean-up and Size Select kit following the manufacturer’s instructions. All samples were pooled to make a master sequencing library. The master library was further purified using gel extraction using the GeneJET Gel Extraction Kit (Thermo Scientific) following the manufacturer’s instructions. All gel extraction solutions were run through a single spin column to maximise DNA yield and concentration was determined using a Collibri^™^ Library Quantification qPCR Kit (Invitrogen). The master sequencing library was run on an Illumina MiSeq sequencer using a 500-cycle paired-end reagent kit (MiSeq Reagent Kits v2,MS-103–2003, Illumina) at a concentration of 15 nM with the addition of 10–15% phiX Control v3 (FC-11–2003, Illumina). FASTQ files were split based on unique indexing barcodes.

### Sequencing analysis

Raw Illumina sequencing data were analysed in R (v4.1.1.) [51] with the DADA2 package v1.20 [52] using the workflow described in nemabiome.ca (https://www.nemabiome.ca/dada2_workflow.html). Reads were trimmed of their primers using Cutadapt v3.4 [53] and any read that did not contain a sequence matching the primer was removed. The remaining reads were trimmed and filtered to leave reads with a minimum length of 200 bp, a maxEE of 6 and truncQ of 2, which then underwent an error learning process within DADA2. For BtA, overlapping forward and reverse reads were merged. The size of the BtB sequenced region was > 600 bp so overlaps were not seen. For BtB, the reads were trimmed to a uniform size of 268 bp and forward and reverse reads were concatenated using “justconcatenate = TRUE” command in DADA2 to merge reads. Finally, chimeras were removed, and the unique sequences were assigned to amplicon sequencing variants (ASVs). The R package phyloseq v1.36 [54] was then used to assign the read counts for each ASV back to the sample data in the form of an Operation Taxonomic Unit (OTU) table.

### Genotyping and population genetics

The ASV sequences were aligned in BioEdit v7.2.5. [55] to check that all ASVs were β-tubulins and any sequences that did not align were removed. Data were screened to remove any read counts below 100 and any sample which had no reads from any ASV was removed. Samples which had reads for more than two ASVs were removed prior to any further analysis. The remaining data were used to screen the ASVs for the common resistance-associated SNPs. Sample information such as population of origin and host species was used to visualise allele frequency distributions in R using ggplot2 v3.3.5. [56]. The allelic richness was calculated for each isotype using the R package PopGenReport v3.0.4. [57].

Data from a recent study which sequenced the same gene regions of *Ascaris* samples from Belgium, Tanzania and Ethiopia were added to the dataset [20]. The DNASP v6.12.03. package [58] was used to create haplotype files. This file included the aligned variable nucleotides only and information on the frequencies of each sequence. PopART v1.7 [59] was used to visualise relationships between ASVs by means of a minimum spanning network. The networks show how each ASV is related to another with dashes representing a single nucleotide change. As haplotype networks can only be made with sequences without gaps, some alleles which differ by only a single insertion or deletion were merged. BtA ASVs 1, 3, 6, 8 and 14 form a single haplotype and in BtB ASVs 2 and 7, ASVs 18 and 24 and ASVs 21 and 29 were merged.

These sequences were further used to generate a maximum likelihood tree with MegaX using the T92 + G model with 1000 bootstraps [60]. Multiple correspondence analysis (MCA) was performed on the combined dataset to assess patterns in variation using the FactoMineR v2.4*.* [61] and factoextra v1.0.7. [62] packages in R.

Samples from regions with fewer than six isolates were removed from the datasets prior to further population genetics analysis using for Arlequin v3.5.2.2. [63], as these populations will be too small to give reliable results. This led to the removal of samples from Nepal and Guatemala and human samples from the UK. Variable nucleotide sequences for each isotype were combined to create a sequence representing individual genotypes. As not all samples had data for both isotypes, some samples were removed from the dataset for this analysis. Arlequin was used to calculate heterozygosity to test for deviation from Hardy-Weinberg equilibrium (HWE), analysis of molecular variance (AMOVA) and fixation indices. Separate analyses were run for each host and populations were grouped by country of origin.

## Results

### No evidence of resistance associated SNPs in *Ascaris*

The sequencing results yielded data from 190 samples with 187 usable results from β-tubulin isotype A (BtA) and 164 from β-tubulin isotype B (BtB). None of the previously described BZ resistance-associated SNPs were identified in the sequence analysis (Additional file 1: Fig. S1 and Fig. S2).

### Genetic variation in *Ascaris* β-tubulin isotypes

After quality checks and filtering of reads, 8 ASVs were identified for BtA and 20 ASVs were found for BtB, indicating a greater genetic diversity in BtB compared to BtA. For both isotypes, most of the sequenced DNA formed part of an intron, with only a small portion at the end of exon 4 and the beginning of exon 5 needed to capture the resistance-associated SNPs. Most variation in BtA was found in the intron and hence was not translated to any changes in the protein sequence. No variation was noted for any BtA ASV in the exon 4 region of the sequence and only one synonymous change was seen in the exon 5 region for ASV 5 (Additional file 1: Fig. S1). In BtB, most variation is also in the intron although there is some variation in both exons for this isotype. There were two synonymous SNPs in exon 4 and four synonymous SNPs in exon 5. ASVs 8, 11 and 14 all had the same four non-synonymous SNPs, with one in exon 4 and three in exon 5. In addition to this, ASV 14 also had two non-synonymous SNPS in exon 4. In total, SNPS were seen in 14 positions in BtA and 37 positions in BtB (Additional file 1: Fig. S2). A total of 69 genotypes were found in 161 samples. The addition of samples from Roose et al. [20] resulted in 14 BtA ASVs, 25 BtB ASVs and 131 genotypes in 370 samples (Additional file 1: Table S1).

**Fig. 1:**
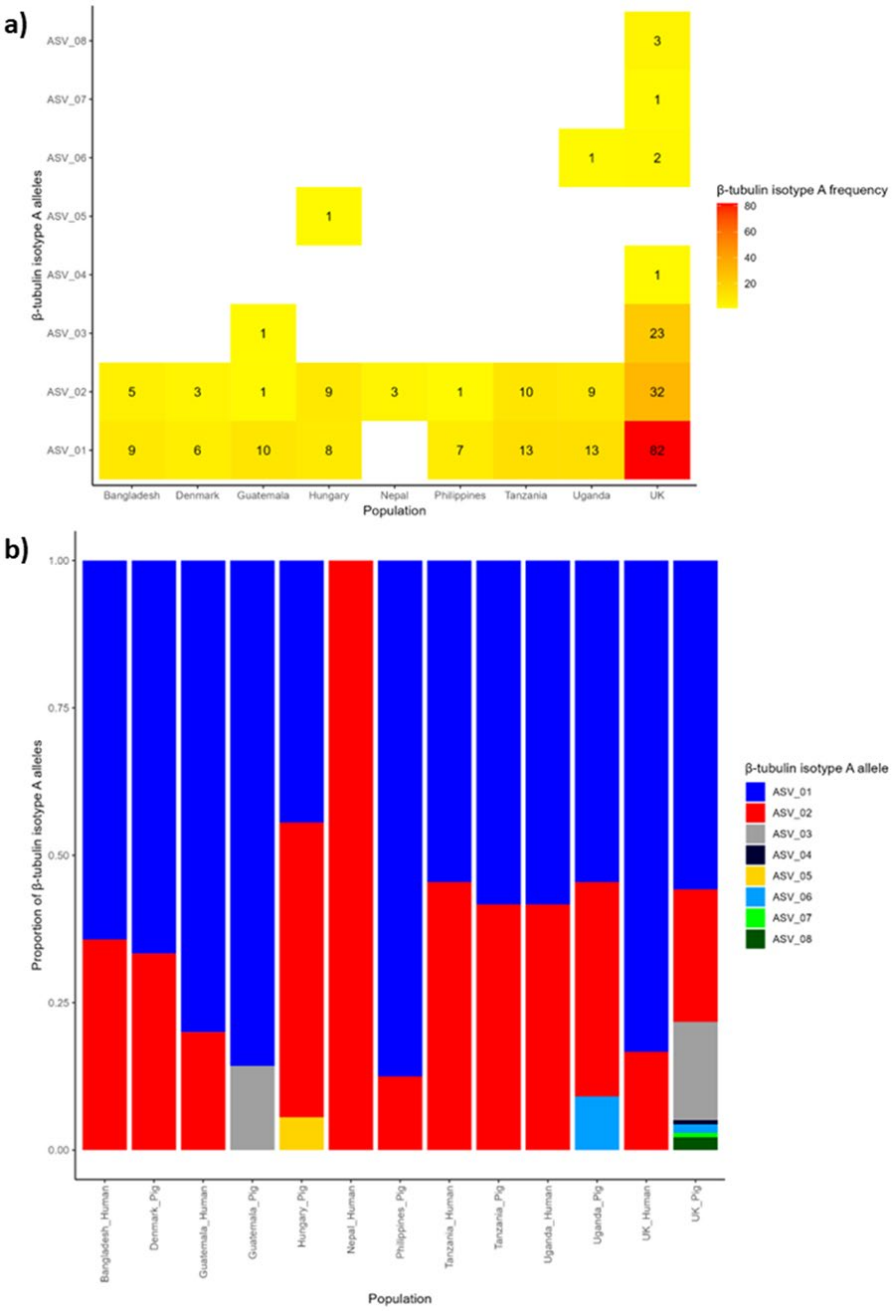
β-tubulin isotype A (BtA) allele frequencies and distribution. **a** Numerical distribution of amplicon sequencing variants (ASVs) among countries; the number of individuals containing each ASV is indicated by the number within tile and tile colour. **b** Representative density of each ASV in the populations separated by country and host species. The 187 samples with data for BtA were used to calculate allele frequencies and distribution

**Fig. 2:**
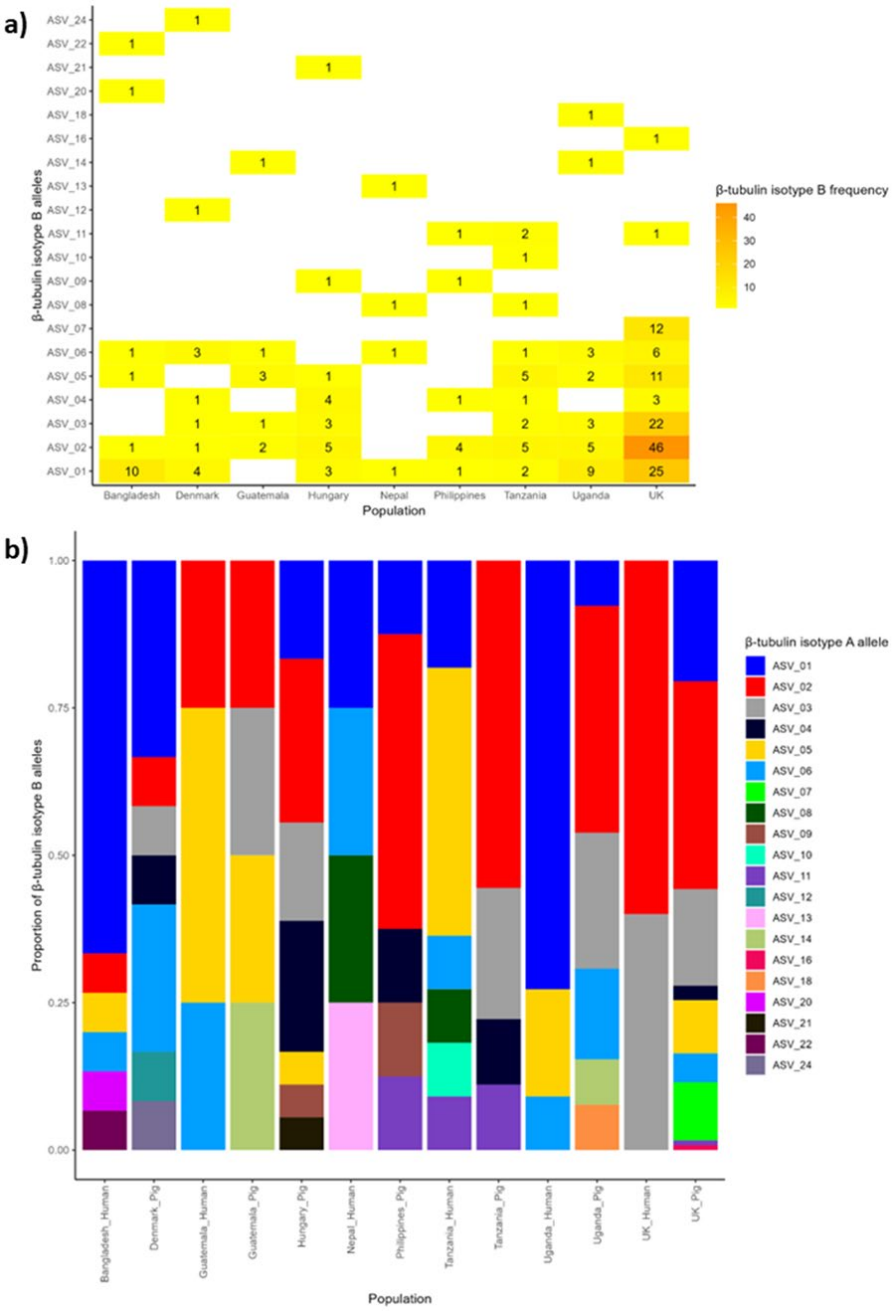
β-Tubulin isotype B (BtB) allele frequencies and distribution. **a** Numerical distribution of amplicon sequencing variants (ASVs) among countries; the number of individuals containing each ASV is indicated by the number within each tile and tile colour. **b** Representative density of each ASV in the populations separated by both country and host species. The 164 samples with data for BtB were used to calculate alleles frequencies and distribution

For BtA, most groups contained only ASVs 1 and 2 when separated by country. Most genetic diversity was seen in the UK samples because of the high proportion of samples originating from this population compared to samples from some countries containing low numbers of ASVs (Fig. 1). For BtB, ASVs 1 to 3 were the most frequently seen across the samples. However, in BtB, ASVs were only found in samples from one or two countries at low frequencies (Fig. 2). The UK pig farms each had their own unique composition of alleles for both β-tubulin isotypes. All the larvae from the UK pig farms A, C and D samples were cultured from eggs isolated from a single faecal sample. Farm B larvae were isolated from multiple faecal samples from a pen housing many piglets. In these samples, BtB was also more diverse than BtA (see Additional file 1: Table S1 for full allele composition).

**Fig. 3:**
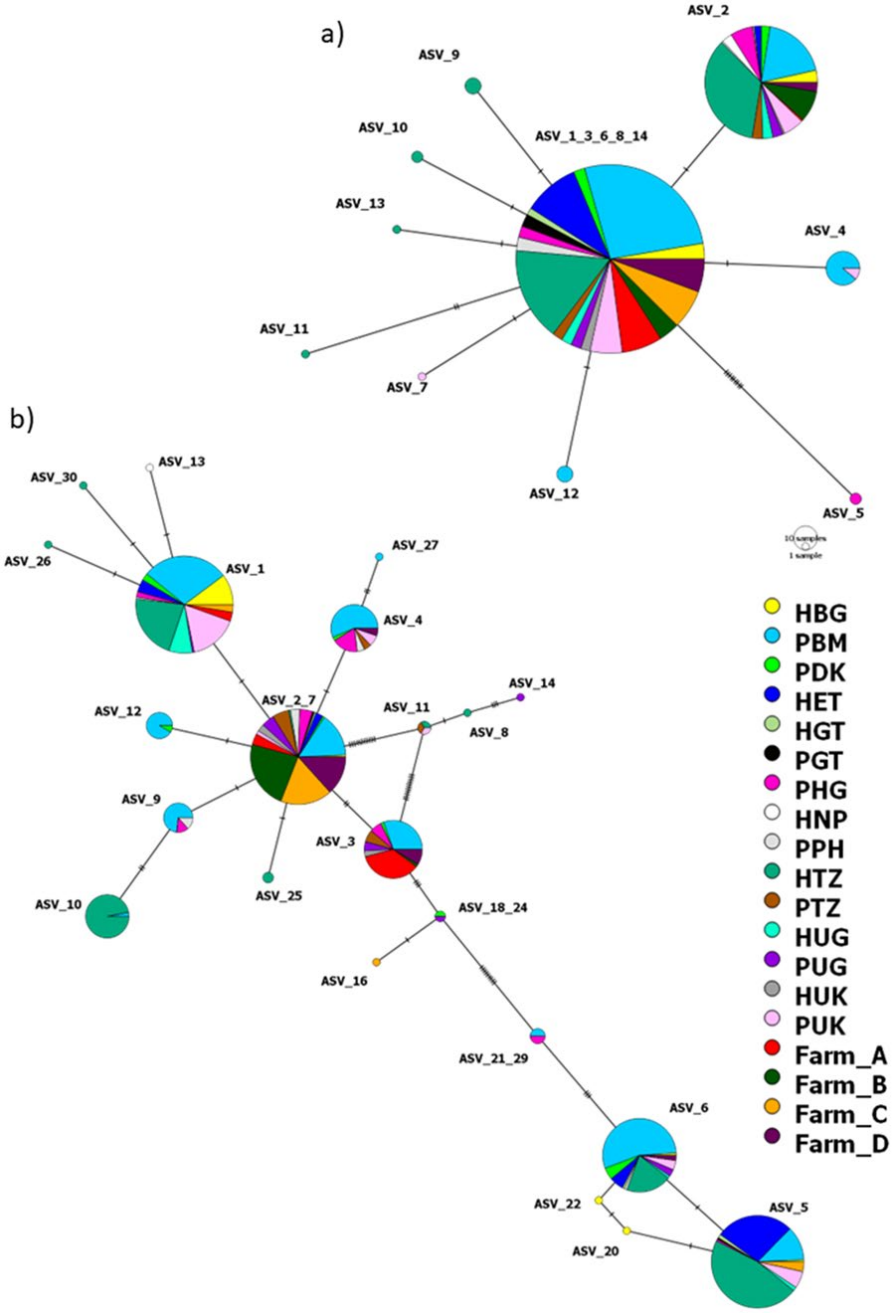
Haplotype distribution. Minimum spanning network showing relationship between amplicon sequencing variant (ASV) with notches between haplotypes representing a sequences change. Each country and host grouping is represented by a unique colour and size of the circle indicates the frequency of the haplotype. There are some regions with numerous changes between two ASVs which is likely due to missing haplotypes that are extinct or yet to be sampled. *H* humans, *P* pig, *BG* Bangladesh, *BM* Belgium, *DK* Denmark, *ET* Ethiopia, *GT* Guatemala, *HG* Hungary, *NP* Nepal, *PH* Philippines, *TZ* Tanzania, *UG* Uganda, *UK* United Kingdom. Farms A, B, C and D indicate the separate pig farms from the UK

The minimum spanning networks showed that BtA ASV1 (ASV_1_3_6_8_14) was the central ASV from which others have diverged (Fig. 3a). For BtA, all ASVs were very similar with only one difference between ASV1 and most of the other ASVs, with the exception of ASV11 with two differences and ASV5 which had nine differences from the central ASV1. Most samples had ASVs 1 and 2 and are comprised of samples from both human and pig hosts (Fig. 3a). For BtB, ASV2 (ASV_2_7) appeared to be the central ASV. However, for BtB, much more divergence was seen between the ASVs, with up to 19 changes seen between the central ASV2 and the most divergent ASVs. Most ASVs contained samples derived from both human and pig hosts in multiple regions so there seems to be no clear structuring; however, ASV4 contains samples from multiple locations that are all of pig origin (Fig. 3b).

The phylogenetic tree created for the combined dataset showed that sequences clustered into three groups (Additional file 1: Fig. S3). Each group contained a mix of samples from both human and pig hosts from different regions.

### Population genetics shows differences between *Ascaris* samples from humans and pigs

The molecular diversity indices showed that the samples from pigs in Belgium and Hungary show significant deviation from HWE for both alleles. Results showed deviation from HWE for BtA in worm samples from humans in Tanzania and pigs in the UK and larval samples from Farm A and Farm B. For BtB, worms from the Philippines and Tanzania and larvae from UK pigs (Farm C and Farm D) deviated from HWE.

A degree of inbreeding within the sample groups was indicated by positive and statistically significant values for the inbreeding coefficient (F_IS_) in the samples from humans in Tanzania and in the pig samples from Belgium, the UK and Farm A (Table 2). AMOVA analysis showed significant variation between different samples within a country and between individuals within a country for each host. Most variation in this analysis was attributed to differences between individuals (Table 3).

**Table 2:** Molecular diversity indices from the combined β-tubulin genotype dataset stratified by host and country

Molecular diversity indices									
Group	Molecular diversity			Hardy-Weinberg equilibrium				Population-specific FIS indices (1023 permutations)	
	Sample size	No. of haplotypes	Average gene diversity over loci	Locus	Obs. Het	Exp. Het	*P*-value	FIS	P(Rand FIS ≥ Obs FIS)
HBG	22	7	0.433	A	0.273	0.455	0.232	0.412	0.226
				B	0.364	0.411	0.451		
HET	58	7	0.305	A	0.069	0.131	0.105	0.477	0.111
				B	0.483	0.479	0.671		
HTZ	184	22	0.626	A	0.250	0.526	< 0.001^a^	0.526	< 0.001^a^
				B	0.696	0.726	0.375		
HUG	16	4	0.421	A	0.500	0.500	1	< 0.001^a^	0.778
				B	0.375	0.342	1		
PBM	208	32	0.652	A	0.269	0.456	< 0.001^a^	0.411	< 0.001^a^
				B	0.750	0.847	0.044^a^		
PDK	14	9	0.632	A	0.286	0.440	0.440	0.368	0.436
				B	0.714	0.824	0.195		
PHG	26	11	0.712	A	0.385	0.588	0.048^a^	0.355	0.096^a^
				B	0.385	0.837	0.000^a^		
PPH	10	4	0.367	A	N/A	N/A	N/A	N/A	N/A
				B	0.200	0.733	0.016^a^		
PTZ	16	6	0.571	A	0.500	0.500	1	< 0.001^a^	0.789
				B	0.125	0.642	0.001^a^		
PUG	16	9	0.675	A	0.375	0.575	0.439	0.364	0.182
				B	0.625	0.775	0.168		
PUK	46	11	0.552	A	0.304	0.486	0.014^a^	0.379	0.025^a^
				B	0.478	0.617	0.107		
Farm A	32	6	0.353	A	0.063	0.179	0.032^a^	0.659	0.029^a^
				B	0.563	0.526	0.580		
Farm B	36	3	0.282	A	0.889	0.508	0.002^a^	-0.167	1
				B	0.056	0.056	1		
Farm C	34	11	0.528	A	0.412	0.355	1	0.252	0.163
				B	0.294	0.701	< 0.001^a^		
Farm D	28	9	0.566	A	0.286	0.378	0.316	-0.789	1
				B	0.357	0.754	0.001^a^		

^a^Indicates a *p*-value < 0.05. Abbreviations: Obs.Het. = observed heterozygosity, Exp.Het. = expected heterozygosity, H = humans, P = pig, BG = Bangladesh, BM = Belgium, DK = Denmark, ET = Ethiopia, GT = Guatemala, HG = Hungary, NP = Nepal, PH = Philippines, TZ = Tanzania, UG = Uganda, UK = United Kingdom. Farms A, B, C and D indicate the separate pig farms from the UK. Hardy-Weinberg equilibrium is calculated for each isotype over 1,000,000 Markov chain steps, and the *p*-value is based on the deviation between the observed and expected values. Population-specific inbreeding coefficient (F_IS_) calculated over 1023 permutations is shown for each population with *p*-values of divergence between random F_IS_ vs observed F_IS_

**Table 3:** Analysis of MOlecular VAriance (AMOVA) to identify sources of variation and calculate fixation indices

Amova analysis								
Host	Source of variation	d.f	Sum of squares	Variance components	Percentage of variation	Fixation indices		*P*-value
Human	Among countries	3	4.989	N/A Va	N/A	FCT	0.000	0.000
	Among individuals within populations	136	44.339	0.10587 Vc	N/A	FIS	0.48088	< 0.001^a^
	Within individuals	140	16	0.11429 Vd	N/A	FIT	0.000	0.000
	Total	279	65.329	N/A				
Pig	Among countries	6	6.156	− 0.06328 Va	-23.85	FCT	− 0.23854	0.69306
	Among individuals within populations	222	60.542	0.05374 Vc	20.26	FIS	0.24541	< 0.001^a^
	Within individuals	233	38.5	0.16524 Vd	62.29	FIT	0.37711	< 0.001^a^
	Total	465	121.605	0.26527				

*Ascaris* samples (individuals) were grouped by country with analysis run separately for worms derived from different hosts. For the UK pig farm data, each farm was designated as a separate population. Results show the degrees of freedom (d.f.) sum of squares and variance components which are used to calculate the percentage of variation contained within the sample divisions. Fixation indices are shown for *F*_CT_, *F*_IS_ and *F*_IT_ along with *p*-values from 1023 permutations

When grouping by worm host, molecular diversity indices showed deviation from HWE and significant F_IS_ in each host (Table 4). AMOVA analysis showed no significant variation between hosts, with low F_ct_ scores indicating low divergence between groups and most variation occurring between individuals or between populations (Table 4). Low *F*_ct_ scores compared to other F-statistics showed that there was more diversity between worms from different countries and regions than there was between worms from different hosts (Table 4). However, both the global and population exact tests of sample differentiation showed that there is a significant difference between the hosts (*p* < 0.001) for both tests.

**Table 4:** Molecular diversity indices based on multiple tests of molecular diversity on the combined β-tubulin genotype dataset

(a) Molecular diversity indices									
	Molecular diversity indices			Hardy-Weinberg equilibrium				Population-specific F_IS_ indices (1023 permutations)	
Group	Sample size	No. of haplotypes	Average gene diversity over loci	Locus	Obs. Het	Exp. Het	*p*-value	*F*_IS_	*p*-value
Human	286	26	0.591	A	0.224	0.468	< 0.001^a^	0.523	< 0.001^a^
				B	0.615	0.714	0.009^a^		
Pig	474	50	0.681	A	0.325	0.517	< 0.001^a^	0.372	< 0.001^a^
				B	0.544	0.845	< 0.001^a^		

(b) Analysis of MOlecular VAriance (AMOVA)							
Source of variation	d.f	Sum of squares	Variance components	Percentage of variation	Fixation indices		*p*-value
Among groups	1	1.587	− 0.0128	− 5.07	F_CT_	− 0.051	0.853
Among populations within groups	17	29.553	0.047	18.84	F_SC_	0.179	< 0.001^a^
Among individuals within populations	361	104.882	0.074	29.23	F_IS_	0.339	< 0.001^a^
Within individuals	380	54.5	0.143	57	F_IT_	0.43	< 0.001^a^

^a^Indicates a *p*-value < 0.05. Abbreviations: Obs.Het. = observed heterozygosity, Exp.Het. = expected heterozygosity. (a) Hardy-Weinberg equilibrium was calculated for each isotype over 1,000,000 Markov chain steps and the *p*-value is based on the deviation between the observed and expected values. Population-specific inbreeding coefficient (F_IS_) calculated over 1023 permutations is shown for each population with *p*-values of divergence between random F_IS_ vs observed F_IS_. (b) Analysis of MOlecular VAriance tests to identify sources of variation and calculate fixation indices. Samples (individuals) were grouped by host species and then populations were separated by country, for the UK pig farm data each farm was designated as a separate population. Samples from humans in the UK were grouped with the pigs as they have been previously shown to be of porcine origin. Results show the degrees of freedom (d.f.) sum of squares and variance components which are used to calculate the percentage of variation contained within the sample divisions. Fixation indices are shown for F_CT_, F_SC_, F_IS_ and F_IT_ along with *p*-values from 1023 permutations. Most variation occurs between individuals within the entire population. Fixation indices show that there is little differentiation between groups but significant differentiation between individuals and populations

**Fig. 4:**
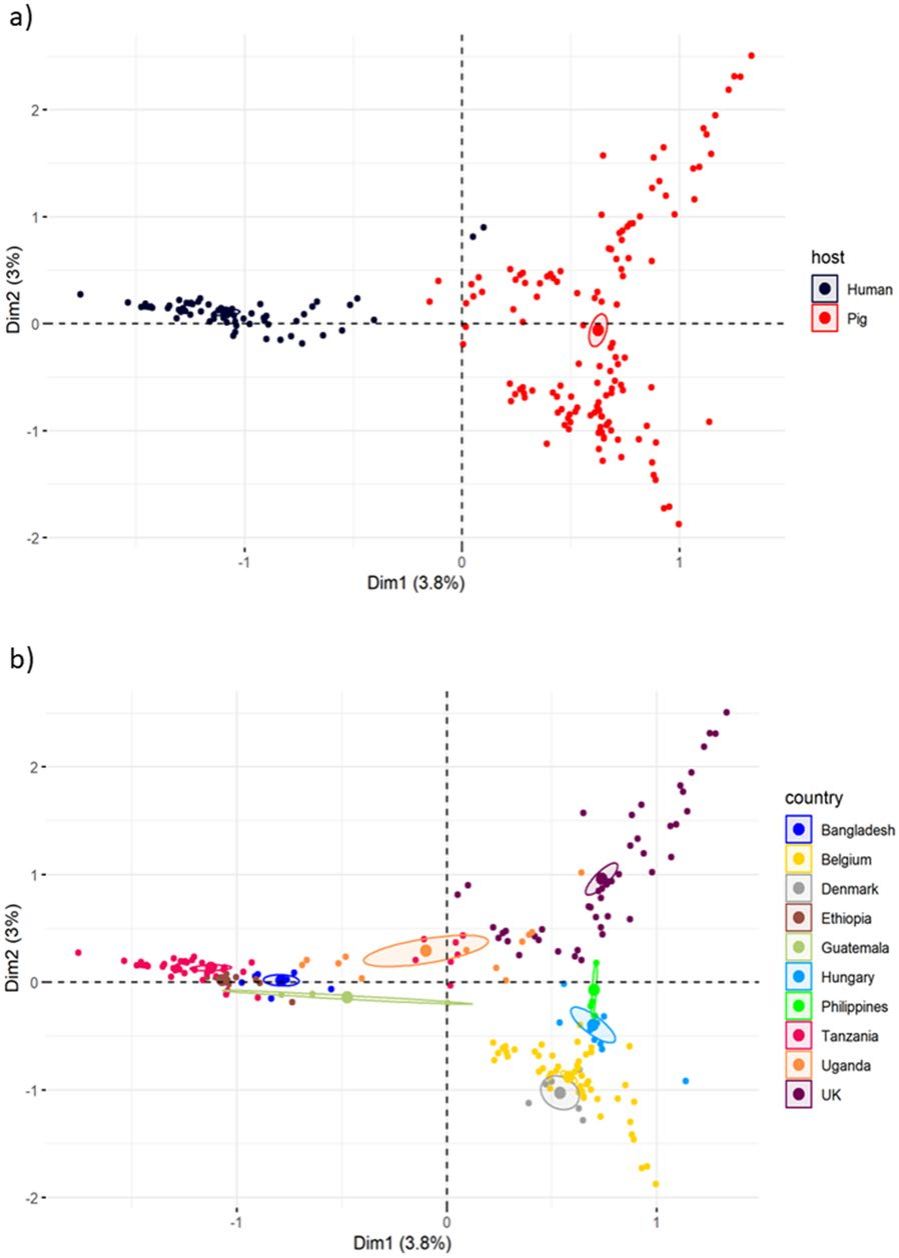
Multiple component analysis (MCA) of *Ascaris* β-tubulins. **a** MCA variance based on host species. **b** Distribution of MCA variance based on country of origin. *H* humans, *P* pig, *BG* Bangladesh, *BM* Belgium, *DK* Denmark, *ET* Ethiopia, *GT* Guatemala, *HG* Hungary, *NP* Nepal, *PH* Philippines, *TZ* Tanzania, *UG* Uganda, *UK* United Kingdom. Farms A, B, C and D indicate the separate pig farms from the UK. Numbers within the population name represent the genotype number

Using initial multiple correspondence analysis (MCA), there was clear separation between the sample hosts, with exception of the UK human samples that clustered closely with the pig samples (Fig. 4a). The MCA showed that there was a greater dispersal of samples of pig origin than there was for the human samples (Fig. 4a). Most samples clustered closely within their country of origin, although for the countries that contained samples from both human and pig hosts, the host does play a role in variation as the samples from different hosts are spread out as seen with the Ugandan and Tanzanian populations (Fig. 4b). Within the pig samples, the continental European samples (Belgium, Denmark and Hungary) clustered close to each other, whereas the Philippine samples were found between the European and UK samples (Fig. 4b).

## Discussion

In this study, an extensive screen of *Ascaris* samples across the world was performed, and we have assessed in detail sequence allelic variation within two β-tubulin isotype genes, showing an absence of canonical resistance-associated SNPs. The inclusion of data from previous studies allowed us to analyse the diversity of these genes in a much larger group of geographically widespread samples, inclusive of human- and pig-derived samples. We found differentiation between *Ascaris* derived from pig and humans, which we consider to indicate a low risk of transfer of resistance genes might they ever arise.

A small number of studies have previously found resistance-associated mutations in *Ascaris*. The F200Y substitution was found at low frequency (0.5%) in Brazil, although, unfortunately, no host treatment history was available so it remains unknown whether these samples were phenotypically resistant to BZs [14]. There were also reports of a high proportion (77–100%) of F167Y substitutions in samples as geographically disparate as Kenya, Haiti and Panama [28]. Even when the F167Y substitutions were present, these populations appeared to have no change in drug susceptibility, and the proportion of these substitutions did not change after treatment, suggesting this mutation alone does not lead to BZ resistance in *Ascaris* [28]. Recent studies looking for evidence of resistance in ascarids have not detected canonical resistance markers, not even in phenotypically resistant parasites [26, 27]. Taken together, the results presented here and in previous studies suggest that BZ resistance via the classical amino acid substitutions in β-tubulins is unlikely to be present in *Ascaris* and closely related nematodes. Whilst not direct orthologues, based on the expression profiles, BtA and BtB in *Ascaris* are analogous to β-tubulin isotypes 1 and 2 in strongyles, respectively [20, 64]. It is important to analyse both of these isotypes when screening for resistance as studies in *H. contortus* have shown that mutations in β-tubulin-2 are also linked to BZ resistance [19].

Our analysis based on variation in BtA and BtB indicated significant differences between *Ascaris* from pigs and *Ascaris* from human hosts, apart from worm samples from humans in the UK, which are likely infections from close contact with pigs and would therefore likely be *A. suum* [39]. This aligns with previous studies [40, 45, 65–67], which found a genetic separation based on host species, but with clear evidence of cross-transmission and recombination due to some shared haplotypes. This indicates that there is a divide between the species, with introgression keeping the diversity low. Thus, if resistance were to arise in human or pig *Ascaris* it might not be able to cross host species barriers.

Our results provide some insights into potential spread of resistance genes between countries and farms. In the MCA analysis, all human-derived samples cluster tightly together. Conversely the pig-derived samples are much more spread with much clearer boundaries based on geographical origin, indicating that there may be greater potential for resistance gene flow between *Ascaris* populations infecting humans than those infecting pigs. At a local level, *Ascaris* samples from all four UK pig farms have unique ASVs which would imply limited population mixing between sites. It should be noted that due to the pooling of larvae prior to extraction, ASVs cannot be assigned to individual larvae in each sample. However, these methods do allow a general overview of the diversity within each farm. With this being said, there is limited diversity within each farm, suggesting that infections may come from the farm environment rather than multiple introductions with stock being brought in. This aligns with the fact that UK farms sampled were all small-scale farms housing various pig breeds from different sources. Studies in Brazil, China, Denmark and the USA found no clear genetic structuring between locations [66–69]. Similarly, a study on *Ascaridia galli* from 10 farms in Denmark and Sweden showed high levels of gene flow between sites [70]. Most farms in Denmark source their pigs from a relatively small number of large-scale breeders resulting in populations of *Ascaris* from those few breeders being spread widely across the country [67]. Thus, the potential for spread of genes associated with anthelmintic resistance between farms depends on the setting and farming practices.

When assessing the relative genetic diversity of human- and pig-derived *Ascaris*, it should be noted that sample sets from each country contained unequal data for each host species, so it is hard to make direct comparisons. The human-derived samples mainly come from regions that have been the target of MDA, which could lead to genetic bottlenecking and a reduced genetic diversity due to the reduced effective population size [71, 72]. Limited information is available about the anthelmintic treatment history of the pig hosts, but two of the UK pig farms where samples were obtained treated pigs with macrocyclic lactones whereas the other two used macrocyclic lactones and BZ.

One limitation of this study is that some of the *Ascaris* samples from humans were obtained after treatment with mebendazole, meaning that they are susceptible to BZs and making it unlikely that they would carry any resistance-associated genes. This contrasts to *Ascaris* samples from pigs which were obtained at slaughter or from faeces. A second limitation is that only two markers (BtA and BtB) were used for population genetic analysis. Use of multiple markers or whole genome comparisons would allow more confident inferences to be made in terms of population genetics. Finally, sample numbers varied substantially between host and location and for samples taken from pigs in UK farms, three out of four sites were composed of samples isolated from a single host meaning that we may not be representing the full diversity of each site.

## Conclusions

Our study adds to the growing evidence that the canonical BZ resistance-associated SNPs seen in other nematodes are not present in *Ascaris* or in other ascarids. This indicates that resistance to BZs in ascarids may depend on an as yet unknown mechanism(s). Future work will need to focus on identifying the possible mechanisms of resistance in the phenotypically resistant populations of ascarids that have been identified and continue to monitor BZ efficacy in ascarids. Meanwhile, the genetic diversity seen in *Ascaris* populations has shown that the parasites infecting pigs and humans represent different populations, suggesting limited potential for flow of resistance genes between *Ascaris* infecting different host species.

### Supplementary Information

**Additional file 1: Fig. S1.** β-Tubulin isotype A (BtA) amplicon sequencing variants (ASVs) sequence alignment. **Fig. S2.** β-Tubulin isotype B (BtB) amplicon sequencing variants (ASVs) sequence alignment. **Fig. S3.** Maximum likelihood phylogeny of *Ascaris* β-tubulin genotypes. **Table S1.** Sample information and β-tubulin isotype alleles.

## Data Availability

All data generated or analysed during this study are included in this published article and its supplementary information files.
