# Supplementary material for: Worldwide absence of canonical benzimidazole resistance-associated mutations within β-tubulin genes from Ascaris
Source: Parasit Vectors. 2024 May 16;17:225. doi: 10.1186/s13071-024-06306-5 (PMC11098727; doi:10.1186/s13071-024-06306-5)
Supplement: Supplementary file 1 — Additional file 1: Fig. S1. β-Tubulin isotype A (BtA) amplicon sequencing variants (ASVs) sequence alignment. Fig. S2. β-Tubulin isotype B (BtB) amplicon sequencing variants (ASVs) sequence alignment. Fig. S3. Maximum likelihood phylogeny of Ascaris β-tubulin genotypes. Table S1. Sample information and β-tubulin isotype alleles. [file 13071_2024_6306_MOESM1_ESM.docx]

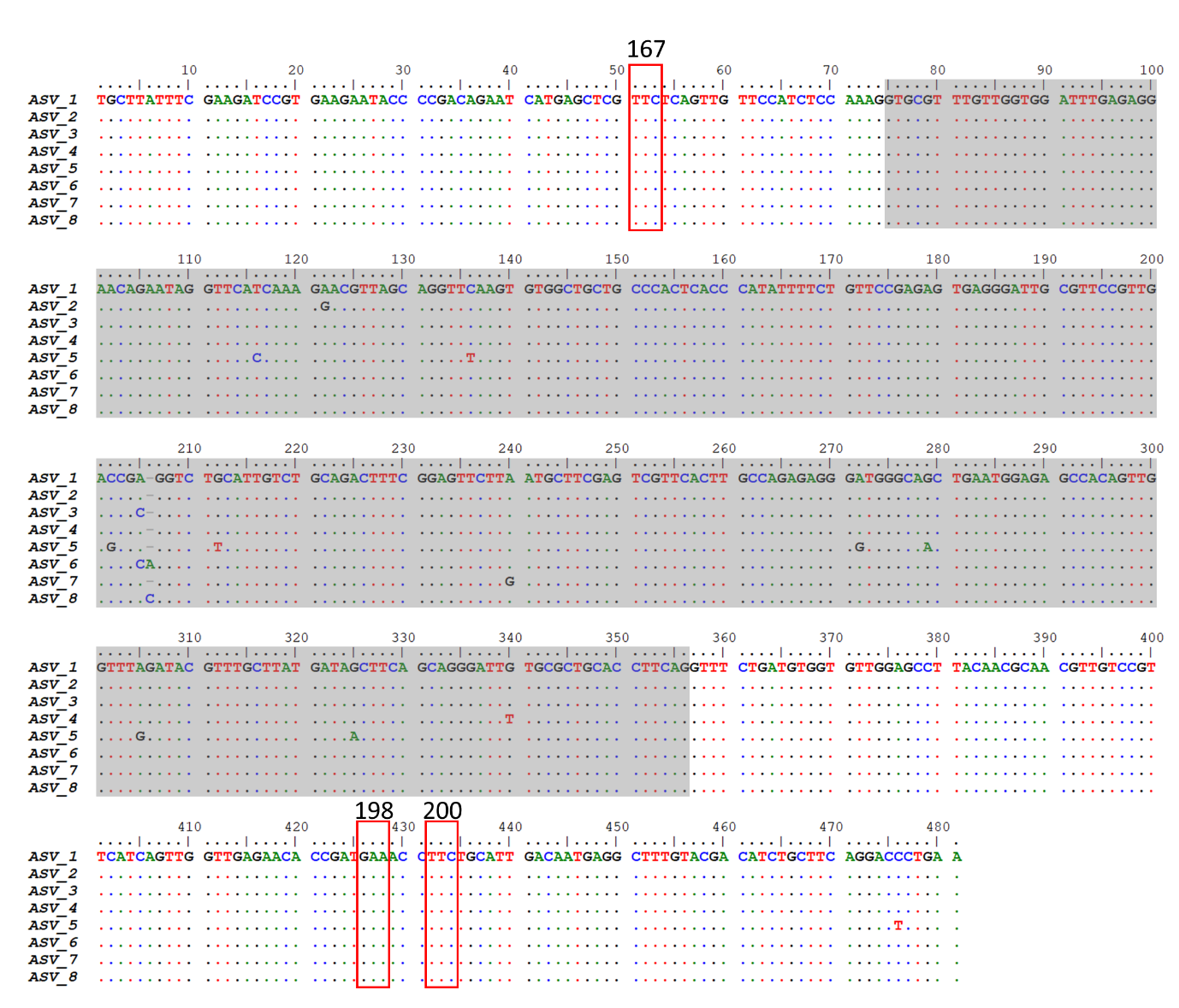


**Fig. S1** β-tubulin isotype A (BtA) amplicon sequencing variants (ASVs) sequence alignment. The alignments show the eight ASVs found for BtA. Nucleotides that are the same as the reference sequence are represented as dots. Codons 167, 198 and 200 are indicated by boxes. The intron has been shaded to illustrate the positions of the SNPs.


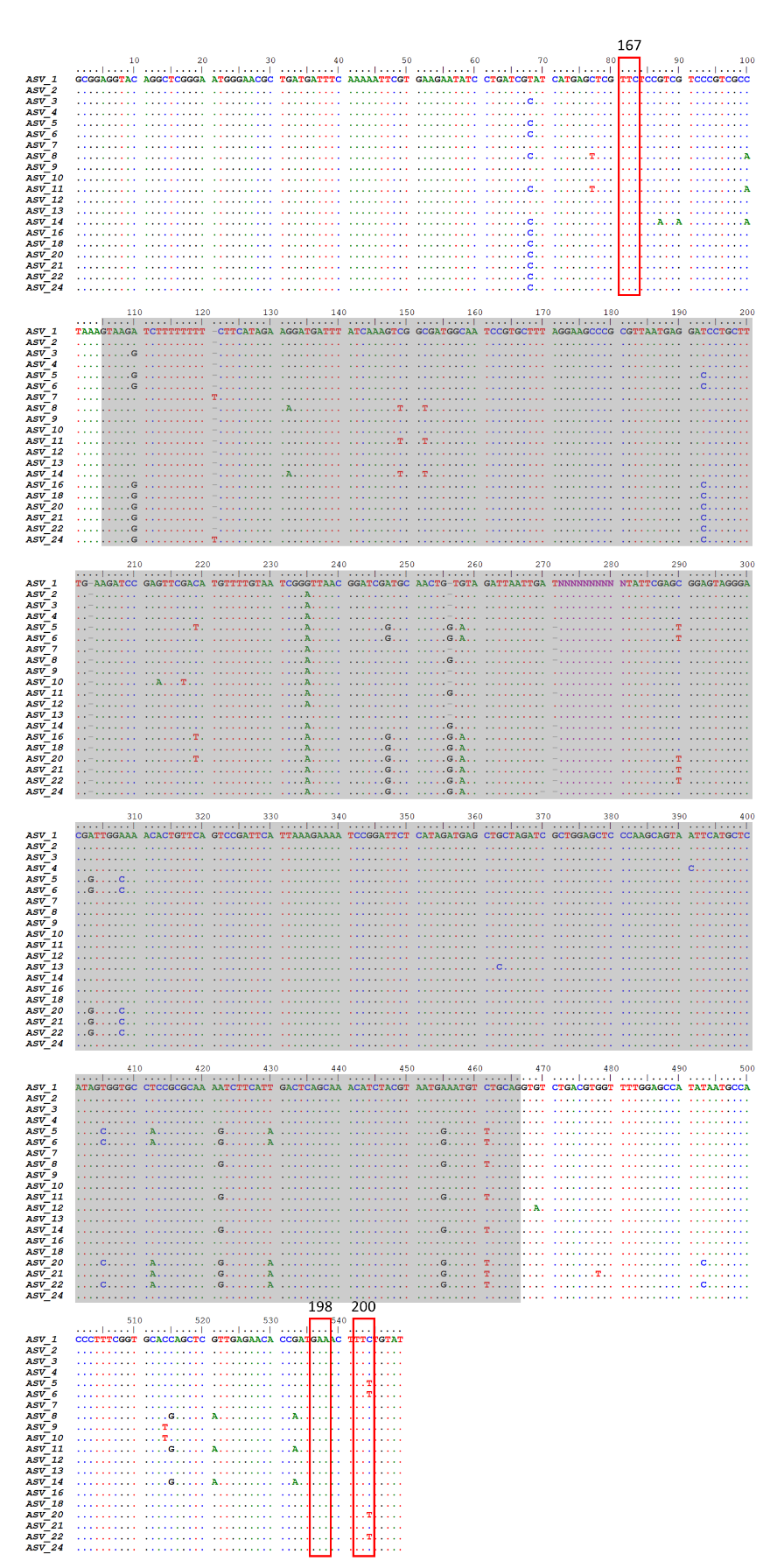


**Fig. S2** β-tubulin isotype B (BtB) amplicon sequencing variants (ASVs) sequence alignment. The alignments show the 20 ASVs found for BtB. Nucleotides that are the same as the reference sequence are represented as dots. Codons 167, 198 and 200 are indicated by boxes. No changes are seen in the 167 and 198 codons however there are four ASVs which have synonymous changes in codon 200. The intron has been shaded to illustrate the positions of the SNPs.

**Table S1.** Sample information and β-tubulin isotype alleles.

| **Sample** | **ID** | **Population** | **Country** | **Region** | **Sample type** | **Host** | **BtA alleles** | **BtB alleles** | **Genotype** |
| --- | --- | --- | --- | --- | --- | --- | --- | --- | --- |
| HBA12.1 | HBA12.1 | HBG | Bangladesh | Unknown | adult | Human | ASV_1/ASV_1 | ASV_1/ASV_1 | 1 |
| HBA15.2 | HBA15.2 | HBG | Bangladesh | Unknown | adult | Human | ASV_1/ASV_1 | ASV_1/ASV_1 | 1 |
| HBA16.1 | HBA16.1 | HBG | Bangladesh | Unknown | adult | Human | ASV_2/ASV_2 | ASV_1/ASV_1 | 50 |
| HBA17.1 | HBA17.1 | HBG | Bangladesh | Unknown | adult | Human | ASV_1/ASV_2 | ASV_1/ASV_5 | 23 |
| HBA19.2 | HBA19.2 | HBG | Bangladesh | Unknown | adult | Human | ASV_1/ASV_1 | ASV_20/ASV_22 | 20 |
| HBA22.1 | HBA22.1 | HBG | Bangladesh | Unknown | adult | Human | ASV_1/ASV_2 | ASV_1/ASV_1 | 21 |
| HBA23.1 | HBA23.1 | HBG | Bangladesh | Unknown | adult | Human | ASV_1/ASV_1 | ASV_1/ASV_1 | 1 |
| HBA5.1 | HBA5.1 | HBG | Bangladesh | Unknown | adult | Human | ASV_1/ASV_2 | ASV_1/ASV_1 | 21 |
| HBA6.1 | HBA6.1 | HBG | Bangladesh | Unknown | adult | Human | ASV_1/ASV_1 | ASV_1/ASV_1 | 1 |
| HBA7.2 | HBA7.2 | HBG | Bangladesh | Unknown | adult | Human | ASV_2/ASV_2 | ASV_1/ASV_6 | 52 |
| HBA8.1 | HBA8.1 | HBG | Bangladesh | Unknown | adult | Human | ASV_1/ASV_1 | ASV_1/ASV_2 | 2 |
| Asu-006 * | PBM6 | PBM | Belgium | Flanders | adult | Pig | ASV_1/ASV_1 | ASV_1/ASV_5 | 5 |
| Asu-007 * | PBM7 | PBM | Belgium | Flanders | adult | Pig | ASV_1/ASV_1 | ASV_1/ASV_12 | 71 |
| Asu-008 * | PBM8 | PBM | Belgium | Flanders | adult | Pig | ASV_2/ASV_2 | ASV_2/ASV_5 | 119 |
| Asu-009 * | PBM9 | PBM | Belgium | Flanders | adult | Pig | ASV_1/ASV_2 | ASV_4/ASV_6 | 95 |
| Asu-010 * | PBM10 | PBM | Belgium | Flanders | adult | Pig | ASV_4/ASV_4 | ASV_1/ASV_4 | 129 |
| Asu-011 * | PBM11 | PBM | Belgium | Flanders | adult | Pig | ASV_1/ASV_1 | ASV_1/ASV_12 | 71 |
| Asu-012 * | PBM12 | PBM | Belgium | Flanders | adult | Pig | ASV_1/ASV_2 | ASV_2/ASV_3 | 28 |
| Asu-013 * | PBM13 | PBM | Belgium | Flanders | adult | Pig | ASV_1/ASV_2 | ASV_1/ASV_1 | 21 |
| Asu-014 * | PBM14 | PBM | Belgium | Flanders | adult | Pig | ASV_1/ASV_2 | ASV_3/ASV_12 | 94 |
| Asu-015 * | PBM15 | PBM | Belgium | Flanders | adult | Pig | ASV_1/ASV_1 | ASV_1/ASV_1 | 1 |
| Asu-016 * | PBM16 | PBM | Belgium | Flanders | adult | Pig | ASV_1/ASV_1 | ASV_1/ASV_1 | 1 |
| Asu-017 * | PBM17 | PBM | Belgium | Flanders | adult | Pig | ASV_1/ASV_2 | ASV_1/ASV_1 | 21 |
| Asu-018 * | PBM18 | PBM | Belgium | Flanders | adult | Pig | ASV_1/ASV_2 | ASV_1/ASV_3 | 22 |
| Asu-019 * | PBM19 | PBM | Belgium | Flanders | adult | Pig | ASV_2/ASV_2 | ASV_1/ASV_3 | 115 |
| Asu-020 * | PBM20 | PBM | Belgium | Flanders | adult | Pig | ASV_1/ASV_1 | ASV_1/ASV_5 | 5 |
| Asu-021 * | PBM21 | PBM | Belgium | Flanders | adult | Pig | ASV_2/ASV_2 | ASV_1/ASV_1 | 50 |
| Asu-022 * | PBM22 | PBM | Belgium | Flanders | adult | Pig | ASV_1/ASV_2 | ASV_1/ASV_6 | 24 |
| Asu-023 * | PBM23 | PBM | Belgium | Flanders | adult | Pig | ASV_1/ASV_1 | ASV_6/ASV_6 | 85 |
| Asu-024 * | PBM24 | PBM | Belgium | Flanders | adult | Pig | ASV_1/ASV_1 | ASV_5/ASV_6 | 15 |
| Asu-025 * | PBM25 | PBM | Belgium | Flanders | adult | Pig | ASV_1/ASV_1 | ASV_1/ASV_6 | 6 |
| Asu-026 * | PBM26 | PBM | Belgium | Flanders | adult | Pig | ASV_1/ASV_1 | ASV_3/ASV_6 | 78 |
| Asu-027 * | PBM27 | PBM | Belgium | Flanders | Adult | Pig | ASV_1/ASV_2 | NA | NA |
| Asu-028 * | PBM28 | PBM | Belgium | Flanders | adult | Pig | ASV_1/ASV_2 | ASV_4/ASV_6 | 95 |
| Asu-031 * | PBM31 | PBM | Belgium | Flanders | adult | Pig | ASV_1/ASV_1 | ASV_2/ASV_6 | 74 |
| Asu-033 * | PBM33 | PBM | Belgium | Flanders | adult | Pig | ASV_1/ASV_1 | ASV_2/ASV_5 | 9 |
| Asu-034 * | PBM34 | PBM | Belgium | Flanders | adult | Pig | ASV_1/ASV_1 | ASV_4/ASV_6 | 79 |
| Asu-036 * | PBM36 | PBM | Belgium | Flanders | adult | Pig | ASV_1/ASV_1 | ASV_1/ASV_12 | 71 |
| Asu-038 * | PBM38 | PBM | Belgium | Flanders | adult | Pig | ASV_1/ASV_1 | ASV_4/ASV_4 | 13 |
| Asu-041 * | PBM41 | PBM | Belgium | Flanders | adult | Pig | ASV_12/ASV_12 | ASV_9/ASV_12 | 110 |
| Asu-044 * | PBM44 | PBM | Belgium | Flanders | adult | Pig | ASV_1/ASV_2 | ASV_1/ASV_6 | 24 |
| Asu-046 * | PBM46 | PBM | Belgium | Flanders | adult | Pig | ASV_1/ASV_2 | ASV_1/ASV_2 | 88 |
| Asu-047 * | PBM47 | PBM | Belgium | Flanders | adult | Pig | ASV_1/ASV_2 | ASV_1/ASV_5 | 23 |
| Asu-050 * | PBM50 | PBM | Belgium | Flanders | adult | Pig | ASV_1/ASV_1 | ASV_4/ASV_6 | 79 |
| Asu-051 * | PBM51 | PBM | Belgium | Flanders | Adult | Pig | ASV_1/ASV_2 | NA | NA |
| Asu-052 * | PBM52 | PBM | Belgium | Flanders | adult | Pig | ASV_1/ASV_1 | ASV_1/ASV_2 | 2 |
| Asu-054 * | PBM54 | PBM | Belgium | Flanders | adult | Pig | ASV_4/ASV_4 | ASV_1/ASV_2 | 128 |
| Asu-056 * | PBM56 | PBM | Belgium | Flanders | adult | Pig | ASV_1/ASV_1 | ASV_1/ASV_5 | 5 |
| Asu-057 * | PBM57 | PBM | Belgium | Flanders | Adult | Pig | ASV_1/ASV_2 | NA | NA |
| Asu-059 * | PBM59 | PBM | Belgium | Flanders | adult | Pig | ASV_1/ASV_1 | ASV_1/ASV_2 | 2 |
| Asu-063 * | PBM63 | PBM | Belgium | Flanders | adult | Pig | ASV_1/ASV_4 | ASV_2/ASV_4 | 105 |
| Asu-064 * | PBM64 | PBM | Belgium | Flanders | adult | Pig | ASV_1/ASV_2 | ASV_1/ASV_6 | 24 |
| Asu-067 * | PBM67 | PBM | Belgium | Flanders | adult | Pig | ASV_1/ASV_1 | ASV_1/ASV_6 | 6 |
| Asu-069 * | PBM69 | PBM | Belgium | Flanders | adult | Pig | ASV_1/ASV_1 | ASV_1/ASV_2 | 2 |
| Asu-072 * | PBM72 | PBM | Belgium | Flanders | adult | Pig | ASV_1/ASV_1 | ASV_1/ASV_4 | 4 |
| Asu-073 * | PBM73 | PBM | Belgium | Flanders | adult | Pig | ASV_4/ASV_4 | ASV_1/ASV_2 | 128 |
| Asu-074 * | PBM74 | PBM | Belgium | Flanders | adult | Pig | ASV_4/ASV_4 | ASV_2/ASV_2 | 130 |
| Asu-076 * | PBM76 | PBM | Belgium | Flanders | adult | Pig | ASV_1/ASV_1 | ASV_1/ASV_6 | 6 |
| Asu-077 * | PBM77 | PBM | Belgium | Flanders | adult | Pig | ASV_1/ASV_1 | ASV_1/ASV_1 | 1 |
| Asu-079 * | PBM79 | PBM | Belgium | Flanders | adult | Pig | ASV_1/ASV_1 | ASV_1/ASV_2 | 2 |
| Asu-080 * | PBM80 | PBM | Belgium | Flanders | adult | Pig | ASV_2/ASV_2 | ASV_4/ASV_5 | 121 |
| Asu-081 * | PBM81 | PBM | Belgium | Flanders | adult | Pig | ASV_1/ASV_1 | ASV_3/ASV_3 | 11 |
| Asu-084 * | PBM84 | PBM | Belgium | Flanders | adult | Pig | ASV_1/ASV_1 | ASV_4/ASV_4 | 13 |
| Asu-093 * | PBM93 | PBM | Belgium | Flanders | adult | Pig | ASV_2/ASV_2 | ASV_2/ASV_4 | 118 |
| Asu-099 * | PBM99 | PBM | Belgium | Flanders | adult | Pig | ASV_4/ASV_4 | ASV_1/ASV_1 | 68 |
| Asu-102 * | PBM102 | PBM | Belgium | Flanders | adult | Pig | ASV_1/ASV_2 | ASV_1/ASV_9 | 89 |
| Asu-106 * | PBM106 | PBM | Belgium | Flanders | adult | Pig | ASV_1/ASV_1 | ASV_6/ASV_6 | 85 |
| Asu-108 * | PBM108 | PBM | Belgium | Flanders | adult | Pig | ASV_2/ASV_2 | ASV_1/ASV_3 | 115 |
| Asu-112 * | PBM112 | PBM | Belgium | Flanders | adult | Pig | ASV_4/ASV_4 | ASV_2/ASV_6 | 131 |
| Asu-113 * | PBM113 | PBM | Belgium | Flanders | adult | Pig | ASV_1/ASV_1 | ASV_3/ASV_12 | 75 |
| Asu-114 * | PBM114 | PBM | Belgium | Flanders | adult | Pig | ASV_1/ASV_1 | ASV_3/ASV_4 | 76 |
| Asu-115 * | PBM115 | PBM | Belgium | Flanders | adult | Pig | ASV_1/ASV_1 | ASV_6/ASV_6 | 85 |
| Asu-116 * | PBM116 | PBM | Belgium | Flanders | adult | Pig | ASV_1/ASV_1 | ASV_1/ASV_6 | 6 |
| Asu-121 * | PBM121 | PBM | Belgium | Flanders | adult | Pig | ASV_1/ASV_1 | ASV_6/ASV_6 | 85 |
| Asu-122 * | PBM122 | PBM | Belgium | Flanders | adult | Pig | ASV_1/ASV_1 | ASV_9/ASV_9 | 18 |
| Asu-123 * | PBM123 | PBM | Belgium | Flanders | adult | Pig | ASV_1/ASV_2 | ASV_9/ASV_12 | 102 |
| Asu-124 * | PBM124 | PBM | Belgium | Flanders | adult | Pig | ASV_1/ASV_4 | ASV_1/ASV_6 | 104 |
| Asu-125 * | PBM125 | PBM | Belgium | Flanders | adult | Pig | ASV_2/ASV_2 | ASV_2/ASV_6 | 120 |
| Asu-126 * | PBM126 | PBM | Belgium | Flanders | adult | Pig | ASV_1/ASV_2 | ASV_6/ASV_7 | 100 |
| Asu-127 * | PBM127 | PBM | Belgium | Flanders | adult | Pig | ASV_1/ASV_2 | ASV_1/ASV_6 | 24 |
| Asu-128 * | PBM128 | PBM | Belgium | Flanders | adult | Pig | ASV_1/ASV_1 | ASV_4/ASV_4 | 13 |
| Asu-129 * | PBM129 | PBM | Belgium | Flanders | adult | Pig | ASV_1/ASV_2 | ASV_6/ASV_6 | 36 |
| Asu-130 * | PBM130 | PBM | Belgium | Flanders | adult | Pig | ASV_1/ASV_1 | ASV_1/ASV_9 | 72 |
| Asu-131 * | PBM131 | PBM | Belgium | Flanders | adult | Pig | ASV_1/ASV_1 | ASV_3/ASV_5 | 77 |
| Asu-132 * | PBM132 | PBM | Belgium | Flanders | adult | Pig | ASV_12/ASV_12 | ASV_6/ASV_12 | 109 |
| Asu-133 * | PBM133 | PBM | Belgium | Flanders | adult | Pig | ASV_1/ASV_2 | ASV_2/ASV_6 | 92 |
| Asu-134 * | PBM134 | PBM | Belgium | Flanders | adult | Pig | ASV_1/ASV_2 | ASV_27/ASV_29 | 93 |
| Asu-135 * | PBM135 | PBM | Belgium | Flanders | adult | Pig | ASV_1/ASV_1 | ASV_2/ASV_6 | 74 |
| Asu-136 * | PBM136 | PBM | Belgium | Flanders | adult | Pig | ASV_1/ASV_1 | ASV_6/ASV_10 | 83 |
| Asu-137 * | PBM137 | PBM | Belgium | Flanders | adult | Pig | ASV_1/ASV_1 | ASV_1/ASV_1 | 1 |
| Asu-138 * | PBM138 | PBM | Belgium | Flanders | adult | Pig | ASV_14/ASV_14 | ASV_2/ASV_12 | 111 |
| Asu-139 * | PBM139 | PBM | Belgium | Flanders | adult | Pig | ASV_1/ASV_1 | ASV_1/ASV_6 | 6 |
| Asu-140 * | PBM140 | PBM | Belgium | Flanders | adult | Pig | ASV_1/ASV_1 | ASV_3/ASV_5 | 77 |
| Asu-141 * | PBM141 | PBM | Belgium | Flanders | adult | Pig | ASV_1/ASV_1 | ASV_4/ASV_6 | 79 |
| Asu-142 * | PBM142 | PBM | Belgium | Flanders | Adult | Pig | ASV_1/ASV_2 | NA | NA |
| Asu-143 * | PBM143 | PBM | Belgium | Flanders | adult | Pig | ASV_1/ASV_2 | ASV_5/ASV_6 | 36 |
| Asu-144 * | PBM144 | PBM | Belgium | Flanders | Adult | Pig | ASV_1/ASV_1 | NA | NA |
| Asu-145 * | PBM145 | PBM | Belgium | Flanders | adult | Pig | ASV_1/ASV_1 | ASV_2/ASV_5 | 9 |
| Asu-146 * | PBM146 | PBM | Belgium | Flanders | adult | Pig | ASV_1/ASV_1 | ASV_6/ASV_29 | 84 |
| Asu-147 * | PBM147 | PBM | Belgium | Flanders | adult | Pig | ASV_1/ASV_1 | ASV_4/ASV_6 | 79 |
| Asu-148 * | PBM148 | PBM | Belgium | Flanders | adult | Pig | ASV_1/ASV_1 | ASV_6/ASV_9 | 86 |
| Asu-149 * | PBM149 | PBM | Belgium | Flanders | adult | Pig | ASV_1/ASV_1 | ASV_6/ASV_6 | 85 |
| Asu-150 * | PBM150 | PBM | Belgium | Flanders | adult | Pig | ASV_1/ASV_2 | ASV_6/ASV_9 | 101 |
| Asu-151 * | PBM151 | PBM | Belgium | Flanders | adult | Pig | ASV_1/ASV_1 | ASV_4/ASV_6 | 79 |
| Asu-152 * | PBM152 | PBM | Belgium | Flanders | adult | Pig | ASV_1/ASV_1 | ASV_3/ASV_12 | 75 |
| Asu-153 * | PBM153 | PBM | Belgium | Flanders | adult | Pig | ASV_1/ASV_2 | ASV_5/ASV_5 | 35 |
| Asu-154 * | PBM154 | PBM | Belgium | Flanders | adult | Pig | ASV_1/ASV_1 | ASV_6/ASV_9 | 86 |
| Asu-155 * | PBM155 | PBM | Belgium | Flanders | adult | Pig | ASV_1/ASV_1 | ASV_3/ASV_3 | 11 |
| Asu-156 * | PBM156 | PBM | Belgium | Flanders | adult | Pig | ASV_1/ASV_4 | ASV_6/ASV_9 | 106 |
| Asu-157 * | PBM157 | PBM | Belgium | Flanders | adult | Pig | ASV_1/ASV_2 | ASV_4/ASV_6 | 95 |
| Asu-158 * | PBM158 | PBM | Belgium | Flanders | adult | Pig | ASV_1/ASV_1 | ASV_4/ASV_5 | 14 |
| Asu-159 * | PBM159 | PBM | Belgium | Flanders | adult | Pig | ASV_1/ASV_1 | ASV_5/ASV_9 | 82 |
| Asu-160 * | PBM160 | PBM | Belgium | Flanders | adult | Pig | ASV_1/ASV_1 | ASV_3/ASV_5 | 77 |
| Asu-161 * | PBM161 | PBM | Belgium | Flanders | adult | Pig | ASV_1/ASV_1 | ASV_5/ASV_6 | 15 |
| Asu-162 * | PBM162 | PBM | Belgium | Flanders | adult | Pig | ASV_1/ASV_2 | ASV_2/ASV_6 | 92 |
| Asu-164 * | PBM164 | PBM | Belgium | Flanders | adult | Pig | ASV_1/ASV_1 | ASV_2/ASV_4 | 73 |
| Asu-165 * | PBM165 | PBM | Belgium | Flanders | adult | Pig | ASV_1/ASV_4 | ASV_1/ASV_3 | 103 |
| Asu-166 * | PBM166 | PBM | Belgium | Flanders | adult | Pig | ASV_1/ASV_1 | ASV_6/ASV_6 | 85 |
| Asu-167 * | PBM167 | PBM | Belgium | Flanders | adult | Pig | ASV_1/ASV_1 | ASV_6/ASV_6 | 85 |
| Asu-168 * | PBM168 | PBM | Belgium | Flanders | adult | Pig | ASV_1/ASV_1 | ASV_3/ASV_12 | 75 |
| PDK22.1 | PDK22.1 | PDK | Denmark | Unknown | adult | Pig | ASV_1/ASV_1 | ASV_1/ASV_6 | 6 |
| PDK24.1 | PDK24.1 | PDK | Denmark | Unknown | adult | Pig | ASV_1/ASV_1 | ASV_1/ASV_3 | 3 |
| PDK25.1 | PDK25.1 | PDK | Denmark | Unknown | adult | Pig | ASV_1/ASV_1 | ASV_1/ASV_4 | 4 |
| PDK26.1 | PDK26.1 | PDK | Denmark | Unknown | adult | Pig | ASV_2/ASV_2 | ASV_1/ASV_24 | 53 |
| PDK27.1 | PDK27.1 | PDK | Denmark | Unknown | adult | Pig | ASV_1/ASV_2 | ASV_6/ASV_6 | 36 |
| PDK28.1 | PDK28.1 | PDK | Denmark | Unknown | adult | Pig | ASV_1/ASV_1 | ASV_2/ASV_12 | 10 |
| PDK29.1 | PDK29.1 | PDK | Denmark | Unknown | adult | Pig | ASV_1/ASV_2 | ASV_6/ASV_6 | 36 |
| Alu-ET020-D4-1 * | HET1 | HET | Ethiopia | Jimma | adult | Human | ASV_1/ASV_1 | ASV_5/ASV_5 | 81 |
| Alu-ET020-D4-2 * | HET2 | HET | Ethiopia | Jimma | adult | Human | ASV_1/ASV_1 | ASV_5/ASV_5 | 81 |
| Alu-ET020-D6 * | HET3 | HET | Ethiopia | Jimma | adult | Human | ASV_1/ASV_1 | ASV_5/ASV_5 | 81 |
| Alu-ET025-D3 * | HET4 | HET | Ethiopia | Jimma | adult | Human | ASV_1/ASV_1 | ASV_1/ASV_5 | 5 |
| Alu-ET025-D4-1 * | HET5 | HET | Ethiopia | Jimma | adult | Human | ASV_1/ASV_1 | ASV_1/ASV_5 | 5 |
| Alu-ET025-D4-2 * | HET6 | HET | Ethiopia | Jimma | adult | Human | ASV_1/ASV_1 | ASV_5/ASV_5 | 81 |
| Alu-ET025-D4-3 * | HET7 | HET | Ethiopia | Jimma | adult | Human | ASV_1/ASV_1 | ASV_5/ASV_5 | 81 |
| Alu-ET025-D5 * | HET8 | HET | Ethiopia | Jimma | adult | Human | ASV_1/ASV_1 | ASV_5/ASV_6 | 15 |
| Alu-ET025-D7-1 * | HET9 | HET | Ethiopia | Jimma | adult | Human | ASV_1/ASV_1 | ASV_5/ASV_6 | 15 |
| Alu-ET025-D7-2 * | HET10 | HET | Ethiopia | Jimma | adult | Human | ASV_1/ASV_1 | ASV_5/ASV_5 | 81 |
| Alu-ET033-D5 * | HET11 | HET | Ethiopia | Jimma | adult | Human | ASV_1/ASV_2 | ASV_1/ASV_5 | 23 |
| Alu-ET061-D3-1 * | HET12 | HET | Ethiopia | Jimma | adult | Human | ASV_1/ASV_1 | ASV_5/ASV_5 | 81 |
| Alu-ET061-D3-3 * | HET13 | HET | Ethiopia | Jimma | adult | Human | ASV_1/ASV_1 | ASV_5/ASV_5 | 81 |
| Alu-ET061-D3-4 * | HET14 | HET | Ethiopia | Jimma | adult | Human | ASV_1/ASV_1 | ASV_5/ASV_6 | 15 |
| Alu-ET061-D3-5 * | HET15 | HET | Ethiopia | Jimma | adult | Human | ASV_1/ASV_1 | ASV_5/ASV_5 | 81 |
| Alu-ET061-D3-6 * | HET16 | HET | Ethiopia | Jimma | adult | Human | ASV_1/ASV_1 | ASV_2/ASV_6 | 74 |
| Alu-ET061-D5 * | HET17 | HET | Ethiopia | Jimma | adult | Human | ASV_1/ASV_1 | ASV_1/ASV_5 | 5 |
| Alu-ET146-D6-1 * | HET18 | HET | Ethiopia | Jimma | adult | Human | ASV_1/ASV_1 | ASV_5/ASV_5 | 81 |
| Alu-ET146-D6-2 * | HET19 | HET | Ethiopia | Jimma | adult | Human | ASV_1/ASV_1 | ASV_5/ASV_6 | 15 |
| Alu-ET151-D5 * | HET20 | HET | Ethiopia | Jimma | adult | Human | ASV_1/ASV_2 | ASV_5/ASV_6 | 36 |
| Alu-ET151-D6 * | HET21 | HET | Ethiopia | Jimma | adult | Human | ASV_1/ASV_1 | ASV_5/ASV_5 | 81 |
| Alu-ET151-D7 * | HET22 | HET | Ethiopia | Jimma | adult | Human | ASV_1/ASV_1 | ASV_5/ASV_5 | 81 |
| Alu-ET220-D5 * | HET23 | HET | Ethiopia | Jimma | adult | Human | ASV_1/ASV_1 | ASV_1/ASV_1 | 1 |
| Alu-ET244-D3-1 * | HET24 | HET | Ethiopia | Jimma | adult | Human | ASV_1/ASV_1 | ASV_5/ASV_5 | 81 |
| Alu-ET244-D3-2 * | HET25 | HET | Ethiopia | Jimma | adult | Human | ASV_1/ASV_1 | ASV_1/ASV_5 | 5 |
| Alu-ET244-D4-1 * | HET26 | HET | Ethiopia | Jimma | adult | Human | ASV_1/ASV_1 | ASV_5/ASV_5 | 81 |
| Alu-ET244-D4-2 * | HET27 | HET | Ethiopia | Jimma | adult | Human | ASV_1/ASV_1 | ASV_2/ASV_5 | 9 |
| Alu-ET244-D4-3 * | HET28 | HET | Ethiopia | Jimma | adult | Human | ASV_2/ASV_2 | ASV_2/ASV_5 | 119 |
| Alu-ET244-D4-4 * | HET29 | HET | Ethiopia | Jimma | adult | Human | ASV_1/ASV_1 | ASV_2/ASV_5 | 9 |
| HGT2.1 | HGT2.1 | HGT | Guatemala | Santa Rosa | adult | Human | ASV_1/ASV_1 | ASV_5/ASV_6 | 15 |
| HGT3.1 | HGT3.1 | HGT | Guatemala | Santa Rosa | adult | Human | ASV_1/ASV_1 | ASV_2/ASV_5 | 9 |
| HGT5.1 | HGT5.1 | HGT | Guatemala | Santa Rosa | Adult | Human | ASV_1/ASV_2 | NA | NA |
| HGT6.1 | HGT6.1 | HGT | Guatemala | Santa Rosa | Adult | Human | ASV_1/ASV_1 | NA | NA |
| PGT1.1 | PGT1.1 | PGT | Guatemala | Santa Rosa | Adult | Pig | ASV_1/ASV_1 | NA | NA |
| PGT2.1 | PGT2.1 | PGT | Guatemala | Santa Rosa | Adult | Pig | ASV_1/ASV_1 | NA | NA |
| PGT3.1 | PGT3.1 | PGT | Guatemala | Santa Rosa | adult | Pig | ASV_1/ASV_1 | ASV_2/ASV_5 | 9 |
| PGT4.1 | PGT4.1 | PGT | Guatemala | Santa Rosa | Adult | Pig | ASV_1/ASV_3 | NA | NA |
| PGT5.1 | PGT5.1 | PGT | Guatemala | Santa Rosa | Adult | Pig | ASV_1/ASV_1 | NA | NA |
| PGT6.1 | PGT6.1 | PGT | Guatemala | Santa Rosa | Adult | Pig | ASV_1/ASV_1 | NA | NA |
| HG1 | HG1 | PHG | Hungary | Forraskut | adult | Pig | ASV_5/ASV_5 | ASV_21/ASV_21 | 69 |
| HG7A | HG7A | PHG | Hungary | Eger | adult | Pig | ASV_1/ASV_2 | ASV_4/ASV_4 | 34 |
| HG7D | HG7D | PHG | Hungary | Eger | adult | Pig | ASV_2/ASV_2 | ASV_2/ASV_2 | 54 |
| HG7E | HG7E | PHG | Hungary | Eger | adult | Pig | ASV_1/ASV_1 | ASV_4/ASV_4 | 13 |
| HG7G | HG7G | PHG | Hungary | Eger | adult | Pig | ASV_2/ASV_2 | ASV_1/ASV_2 | 51 |
| HG7H | HG7H | PHG | Hungary | Eger | adult | Pig | ASV_1/ASV_2 | ASV_3/ASV_5 | 32 |
| HG7I | HG7I | PHG | Hungary | Eger | adult | Pig | ASV_1/ASV_2 | ASV_2/ASV_4 | 29 |
| HG7J | HG7J | PHG | Hungary | Eger | adult | Pig | ASV_1/ASV_2 | ASV_1/ASV_3 | 22 |
| HG7K | HG7K | PHG | Hungary | Eger | adult | Pig | ASV_2/ASV_2 | ASV_2/ASV_2 | 54 |
| HG7L | HG7L | PHG | Hungary | Eger | adult | Pig | ASV_1/ASV_2 | ASV_4/ASV_4 | 34 |
| HG7M | HG7M | PHG | Hungary | Eger | adult | Pig | ASV_1/ASV_1 | ASV_3/ASV_3 | 11 |
| HG7N | HG7N | PHG | Hungary | Eger | adult | Pig | ASV_2/ASV_2 | ASV_1/ASV_2 | 51 |
| HG7O | HG7O | PHG | Hungary | Eger | adult | Pig | ASV_1/ASV_1 | ASV_9/ASV_9 | 18 |
| HNP1.1 | HNP1.1 | HNP | Nepal | Unknown | Adult | Human | ASV_2/ASV_2 | NA | NA |
| HNP2.1 | HNP2.1 | HNP | Nepal | Unknown | adult | Human | ASV_2/ASV_2 | ASV_6/ASV_13 | 57 |
| HNP4.1 | HNP4.1 | HNP | Nepal | Unknown | Adult | Human | ASV_2/ASV_2 | NA | NA |
| PPH1 | PPH1 | PPH | Philippines | Los Baños | adult | Pig | ASV_1/ASV_1 | ASV_4/ASV_4 | 13 |
| PPH2 | PPH2 | PPH | Philippines | Los Baños | Adult | Pig | ASV_1/ASV_1 | NA | NA |
| PPH4 | PPH4 | PPH | Philippines | Los Baños | Adult | Pig | ASV_1/ASV_2 | NA | NA |
| ZB1 | ZB1 | PPH | Philippines | Buwan | adult | Pig | ASV_1/ASV_1 | ASV_2/ASV_2 | 7 |
| ZB2 | ZB2 | PPH | Philippines | Buwan | adult | Pig | ASV_1/ASV_1 | ASV_1/ASV_2 | 2 |
| ZT1 | ZT1 | PPH | Philippines | Trento | adult | Pig | ASV_1/ASV_1 | ASV_2/ASV_2 | 7 |
| ZT2 | ZT2 | PPH | Philippines | Trento | adult | Pig | ASV_1/ASV_1 | ASV_9/ASV_9 | 18 |
| Alu-TA001-D6 * | HTA1 | HTA | Tanzania | Pemba island | adult | Human | ASV_1/ASV_1 | ASV_5/ASV_10 | 80 |
| Alu-TA003-D6 * | HTA2 | HTA | Tanzania | Pemba island | adult | Human | ASV_2/ASV_2 | ASV_5/ASV_10 | 122 |
| Alu-TA004-D5 * | HTA3 | HTA | Tanzania | Pemba island | adult | Human | ASV_2/ASV_2 | ASV_5/ASV_10 | 122 |
| Alu-TA005-D3 * | HTA4 | HTA | Tanzania | Pemba island | adult | Human | ASV_1/ASV_1 | ASV_5/ASV_6 | 15 |
| Alu-TA006-D5 * | HTA5 | HTA | Tanzania | Pemba island | adult | Human | ASV_2/ASV_2 | ASV_5/ASV_10 | 122 |
| Alu-TA007-D2 * | HTA6 | HTA | Tanzania | Pemba island | adult | Human | ASV_1/ASV_1 | ASV_1/ASV_5 | 5 |
| Alu-TA007-D5 * | HTA7 | HTA | Tanzania | Pemba island | adult | Human | ASV_2/ASV_2 | ASV_5/ASV_5 | 55 |
| Alu-TA008-D3 * | HTA8 | HTA | Tanzania | Pemba island | adult | Human | ASV_1/ASV_2 | ASV_5/ASV_10 | 96 |
| Alu-TA008-D5 * | HTA9 | HTA | Tanzania | Pemba island | adult | Human | ASV_1/ASV_1 | ASV_5/ASV_5 | 81 |
| Alu-TA008-D7 * | HTA10 | HTA | Tanzania | Pemba island | adult | Human | ASV_2/ASV_2 | ASV_6/ASV_10 | 125 |
| Alu-TA009-D6 * | HTA11 | HTA | Tanzania | Pemba island | adult | Human | ASV_2/ASV_2 | ASV_5/ASV_6 | 124 |
| Alu-TA010-D7 * | HTA12 | HTA | Tanzania | Pemba island | adult | Human | ASV_1/ASV_2 | ASV_5/ASV_5 | 35 |
| Alu-TA011-D4 * | HTA13 | HTA | Tanzania | Pemba island | adult | Human | ASV_1/ASV_1 | ASV_5/ASV_5 | 81 |
| Alu-TA011-D5 * | HTA14 | HTA | Tanzania | Pemba island | adult | Human | ASV_1/ASV_1 | ASV_5/ASV_10 | 80 |
| Alu-TA012-D6 * | HTA15 | HTA | Tanzania | Pemba island | adult | Human | ASV_1/ASV_2 | ASV_5/ASV_25 | 97 |
| Alu-TA014-D2 * | HTA16 | HTA | Tanzania | Pemba island | adult | Human | ASV_1/ASV_1 | ASV_5/ASV_5 | 81 |
| Alu-TA015-D6 * | HTA17 | HTZ | Tanzania | Pemba island | Adult | Human | ASV_1/ASV_1 | NA | NA |
| Alu-TA017-D7 * | HTA18 | HTA | Tanzania | Pemba island | adult | Human | ASV_2/ASV_2 | ASV_6/ASV_10 | 125 |
| Alu-TA018-D1 * | HTA19 | HTA | Tanzania | Pemba island | adult | Human | ASV_2/ASV_2 | ASV_10/ASV_10 | 117 |
| Alu-TA019-D4 * | HTA20 | HTA | Tanzania | Pemba island | adult | Human | ASV_1/ASV_1 | ASV_5/ASV_5 | 81 |
| Alu-TA020-D3 * | HTA21 | HTA | Tanzania | Pemba island | adult | Human | ASV_2/ASV_2 | ASV_1/ASV_10 | 114 |
| Alu-TA021-D4 * | HTA22 | HTA | Tanzania | Pemba island | adult | Human | ASV_1/ASV_1 | ASV_1/ASV_5 | 5 |
| Alu-TA021-D5 * | HTA23 | HTA | Tanzania | Pemba island | adult | Human | ASV_2/ASV_2 | ASV_6/ASV_6 | 126 |
| Alu-TA022-D6 * | HTA24 | HTA | Tanzania | Pemba island | adult | Human | ASV_2/ASV_13 | ASV_1/ASV_10 | 113 |
| Alu-TA023-D4 * | HTA25 | HTA | Tanzania | Pemba island | adult | Human | ASV_1/ASV_2 | ASV_1/ASV_5 | 23 |
| Alu-TA025-D5 * | HTA26 | HTA | Tanzania | Pemba island | adult | Human | ASV_1/ASV_1 | ASV_6/ASV_6 | 85 |
| Alu-TA026-D4 * | HTA27 | HTA | Tanzania | Pemba island | adult | Human | ASV_1/ASV_2 | ASV_5/ASV_5 | 35 |
| Alu-TA026-D6 * | HTA28 | HTA | Tanzania | Pemba island | adult | Human | ASV_2/ASV_11 | ASV_1/ASV_1 | 112 |
| Alu-TA028-D6 * | HTA29 | HTA | Tanzania | Pemba island | adult | Human | ASV_1/ASV_2 | ASV_1/ASV_10 | 87 |
| Alu-TA029-D5 * | HTA30 | HTA | Tanzania | Pemba island | adult | Human | ASV_1/ASV_2 | ASV_5/ASV_30 | 99 |
| Alu-TA030-D4 * | HTA31 | HTA | Tanzania | Pemba island | adult | Human | ASV_1/ASV_1 | ASV_1/ASV_5 | 5 |
| Alu-TA031-D3 * | HTA32 | HTA | Tanzania | Pemba island | adult | Human | ASV_1/ASV_2 | ASV_1/ASV_5 | 23 |
| Alu-TA031-D6 * | HTA33 | HTA | Tanzania | Pemba island | adult | Human | ASV_1/ASV_1 | ASV_6/ASV_6 | 85 |
| Alu-TA032-D3 * | HTA34 | HTA | Tanzania | Pemba island | adult | Human | ASV_1/ASV_1 | ASV_5/ASV_10 | 80 |
| Alu-TA034-D4-1 * | HTA35 | HTA | Tanzania | Pemba island | adult | Human | ASV_2/ASV_2 | ASV_10/ASV_10 | 117 |
| Alu-TA034-D4-2 * | HTA36 | HTA | Tanzania | Pemba island | adult | Human | ASV_10/ASV_10 | ASV_1/ASV_5 | 108 |
| Alu-TA036-D4 * | HTA37 | HTA | Tanzania | Pemba island | adult | Human | ASV_1/ASV_1 | ASV_6/ASV_6 | 85 |
| Alu-TA036-D5 * | HTA38 | HTA | Tanzania | Pemba island | adult | Human | ASV_1/ASV_1 | ASV_1/ASV_5 | 5 |
| Alu-TA036-D6 * | HTA39 | HTA | Tanzania | Pemba island | adult | Human | ASV_1/ASV_1 | ASV_5/ASV_5 | 81 |
| Alu-TA038-D4 * | HTA40 | HTA | Tanzania | Pemba island | adult | Human | ASV_1/ASV_2 | ASV_1/ASV_5 | 23 |
| Alu-TA039-D2 * | HTA41 | HTA | Tanzania | Pemba island | adult | Human | ASV_1/ASV_1 | ASV_1/ASV_10 | 70 |
| Alu-TA039-D3 * | HTA42 | HTA | Tanzania | Pemba island | adult | Human | ASV_1/ASV_1 | ASV_5/ASV_10 | 80 |
| Alu-TA040-D4 * | HTA43 | HTA | Tanzania | Pemba island | adult | Human | ASV_2/ASV_2 | ASV_5/ASV_5 | 55 |
| Alu-TA040-D5 * | HTA44 | HTA | Tanzania | Pemba island | adult | Human | ASV_1/ASV_9 | ASV_1/ASV_1 | 107 |
| Alu-TA041-D4 * | HTA45 | HTA | Tanzania | Pemba island | adult | Human | ASV_1/ASV_1 | ASV_5/ASV_5 | 81 |
| Alu-TA043-D6 * | HTA46 | HTA | Tanzania | Pemba island | adult | Human | ASV_1/ASV_1 | ASV_5/ASV_10 | 80 |
| Alu-TA044-D3 * | HTA47 | HTA | Tanzania | Pemba island | adult | Human | ASV_1/ASV_2 | ASV_5/ASV_10 | 96 |
| Alu-TA044-D5 * | HTA48 | HTA | Tanzania | Pemba island | adult | Human | ASV_2/ASV_2 | ASV_1/ASV_6 | 52 |
| Alu-TA045-D5 * | HTA49 | HTA | Tanzania | Pemba island | adult | Human | ASV_2/ASV_2 | ASV_5/ASV_25 | 123 |
| Alu-TA046-D7 * | HTA50 | HTA | Tanzania | Pemba island | adult | Human | ASV_1/ASV_1 | ASV_1/ASV_5 | 5 |
| Alu-TA049-D4 * | HTA51 | HTA | Tanzania | Pemba island | adult | Human | ASV_1/ASV_1 | ASV_1/ASV_5 | 5 |
| Alu-TA050-D3 * | HTA52 | HTA | Tanzania | Pemba island | adult | Human | ASV_1/ASV_2 | ASV_5/ASV_10 | 96 |
| Alu-TA051-D4 * | HTA53 | HTA | Tanzania | Pemba island | adult | Human | ASV_1/ASV_1 | ASV_5/ASV_5 | 81 |
| Alu-TA052-D3 * | HTA54 | HTA | Tanzania | Pemba island | adult | Human | ASV_1/ASV_2 | ASV_5/ASV_26 | 98 |
| Alu-TA053-D4 * | HTA55 | HTA | Tanzania | Pemba island | adult | Human | ASV_9/ASV_9 | ASV_1/ASV_5 | 132 |
| Alu-TA053-D5-1 * | HTA56 | HTA | Tanzania | Pemba island | adult | Human | ASV_2/ASV_2 | ASV_1/ASV_6 | 52 |
| Alu-TA053-D5-2 * | HTA57 | HTA | Tanzania | Pemba island | adult | Human | ASV_1/ASV_1 | ASV_1/ASV_1 | 1 |
| Alu-TA053-D5-3 * | HTA58 | HTA | Tanzania | Pemba island | adult | Human | ASV_1/ASV_2 | ASV_2/ASV_28 | 91 |
| Alu-TA053-D6-1 * | HTA59 | HTA | Tanzania | Pemba island | adult | Human | ASV_1/ASV_1 | ASV_1/ASV_5 | 5 |
| Alu-TA053-D6-2 * | HTA60 | HTA | Tanzania | Pemba island | adult | Human | ASV_1/ASV_1 | ASV_1/ASV_5 | 5 |
| Alu-TA053-D6-3 * | HTA61 | HTA | Tanzania | Pemba island | adult | Human | ASV_1/ASV_1 | ASV_6/ASV_10 | 83 |
| Alu-TA053-D6-4 * | HTA62 | HTA | Tanzania | Pemba island | adult | Human | ASV_2/ASV_2 | ASV_1/ASV_5 | 116 |
| Alu-TA053-D6-5 * | HTA63 | HTA | Tanzania | Pemba island | adult | Human | ASV_2/ASV_2 | ASV_1/ASV_10 | 114 |
| Alu-TA053-D6-6 * | HTA64 | HTA | Tanzania | Pemba island | adult | Human | ASV_1/ASV_1 | ASV_1/ASV_5 | 5 |
| Alu-TA053-D7 * | HTA65 | HTA | Tanzania | Pemba island | adult | Human | ASV_1/ASV_2 | ASV_5/ASV_5 | 35 |
| Alu-TA055-D3 * | HTA66 | HTA | Tanzania | Pemba island | adult | Human | ASV_1/ASV_2 | ASV_10/ASV_10 | 90 |
| Alu-TA056-D5 * | HTA67 | HTA | Tanzania | Pemba island | adult | Human | ASV_2/ASV_2 | ASV_5/ASV_5 | 55 |
| Alu-TA057-D2 * | HTA68 | HTA | Tanzania | Pemba island | adult | Human | ASV_1/ASV_1 | ASV_1/ASV_10 | 70 |
| Alu-TA058-D6 * | HTA69 | HTA | Tanzania | Pemba island | adult | Human | ASV_1/ASV_2 | ASV_5/ASV_6 | 36 |
| Alu-TA060-D5 * | HTA70 | HTA | Tanzania | Pemba island | adult | Human | ASV_2/ASV_2 | ASV_10/ASV_10 | 117 |
| Alu-TA061-D6 * | HTA71 | HTA | Tanzania | Pemba island | adult | Human | ASV_1/ASV_1 | ASV_5/ASV_10 | 80 |
| Alu-TA062-D6 * | HTA72 | HTA | Tanzania | Pemba island | adult | Human | ASV_1/ASV_1 | ASV_1/ASV_5 | 5 |
| Alu-TA063-D5 * | HTA73 | HTA | Tanzania | Pemba island | adult | Human | ASV_2/ASV_9 | ASV_5/ASV_6 | 127 |
| Alu-TA065-D7 * | HTA74 | HTA | Tanzania | Pemba island | adult | Human | ASV_2/ASV_2 | ASV_5/ASV_10 | 122 |
| Alu-TA067-D5 * | HTA75 | HTA | Tanzania | Pemba island | adult | Human | ASV_2/ASV_2 | ASV_1/ASV_1 | 50 |
| Alu-TA068-D2 * | HTA76 | HTA | Tanzania | Pemba island | adult | Human | ASV_1/ASV_1 | ASV_1/ASV_5 | 5 |
| Alu-TA068-D4 * | HTA77 | HTA | Tanzania | Pemba island | adult | Human | ASV_2/ASV_2 | ASV_1/ASV_6 | 52 |
| G2 | G2 | HTZ | Tanzania | Unguja | adult | Human | ASV_1/ASV_1 | ASV_10/ASV_10 | 19 |
| G3 | G3 | HTZ | Tanzania | Unguja | Adult | Human | ASV_1/ASV_1 | NA | NA |
| KA5 | KA5 | HTZ | Tanzania | Unguja | adult | Human | ASV_1/ASV_2 | ASV_1/ASV_1 | 21 |
| KA6 | KA6 | HTZ | Tanzania | Unguja | adult | Human | ASV_1/ASV_2 | ASV_5/ASV_6 | 36 |
| KI3 | KI3 | HTZ | Tanzania | Unguja | Adult | Human | ASV_1/ASV_1 | NA | NA |
| KI6 | KI6 | HTZ | Tanzania | Unguja | adult | Human | ASV_2/ASV_2 | ASV_5/ASV_5 | 55 |
| PTZ12.1 | PTZ12.1 | PTZ | Tanzania | Unknown | adult | Pig | ASV_1/ASV_2 | ASV_3/ASV_3 | 31 |
| PTZ12.2 | PTZ12.2 | PTZ | Tanzania | Unknown | adult | Pig | ASV_1/ASV_2 | ASV_2/ASV_11 | 30 |
| PTZ13.1 | PTZ13.1 | PTZ | Tanzania | Unknown | adult | Pig | ASV_1/ASV_1 | ASV_4/ASV_4 | 13 |
| PTZ16.1 | PTZ16.1 | PTZ | Tanzania | Unknown | adult | Pig | ASV_1/ASV_2 | ASV_2/ASV_2 | 27 |
| PTZ4.1 | PTZ4.1 | PTZ | Tanzania | Unknown | adult | Pig | ASV_2/ASV_2 | ASV_2/ASV_2 | 54 |
| PTZ7.2 | PTZ7.2 | PTZ | Tanzania | Unknown | adult | Pig | ASV_1/ASV_1 | ASV_3/ASV_3 | 11 |
| PTZ8.1 | PTZ8.1 | PTZ | Tanzania | Unknown | adult | Pig | ASV_1/ASV_2 | ASV_2/ASV_2 | 27 |
| PTZ9.1 | PTZ9.1 | PTZ | Tanzania | Unknown | adult | Pig | ASV_1/ASV_1 | ASV_2/ASV_2 | 7 |
| TJ7 | TJ7 | HTZ | Tanzania | Unguja | adult | Human | ASV_1/ASV_2 | ASV_5/ASV_11 | 37 |
| TJ8 | TJ8 | HTZ | Tanzania | Unguja | adult | Human | ASV_2/ASV_2 | ASV_5/ASV_8 | 56 |
| HUG2.1 | HUG2.1 | HUG | Uganda | Kabale | adult | Human | ASV_1/ASV_2 | ASV_1/ASV_1 | 21 |
| HUG2.2 | HUG2.2 | HUG | Uganda | Kabale | adult | Human | ASV_1/ASV_1 | ASV_1/ASV_5 | 5 |
| HUG2.3 | HUG2.3 | HUG | Uganda | Kabale | adult | Human | ASV_2/ASV_2 | ASV_1/ASV_1 | 50 |
| HUG2.4 | HUG2.4 | HUG | Uganda | Kabale | adult | Human | ASV_1/ASV_2 | ASV_1/ASV_1 | 21 |
| HUG2.5 | HUG2.5 | HUG | Uganda | Kabale | adult | Human | ASV_1/ASV_1 | ASV_1/ASV_5 | 5 |
| HUG24.1 | HUG24.1 | HUG | Uganda | Kabale | adult | Human | ASV_1/ASV_2 | ASV_1/ASV_1 | 21 |
| HUG26.1 | HUG26.1 | HUG | Uganda | Kabale | adult | Human | ASV_1/ASV_2 | ASV_1/ASV_6 | 24 |
| HUG3.1 | HUG3.1 | HUG | Uganda | Kabale | adult | Human | ASV_1/ASV_1 | ASV_1/ASV_1 | 1 |
| KLA1 | KLA1 | PUG | Uganda | Kampala | adult | Pig | ASV_2/ASV_2 | ASV_1/ASV_2 | 51 |
| KLA2 | KLA2 | PUG | Uganda | Kampala | adult | Pig | ASV_1/ASV_2 | ASV_6/ASV_6 | 36 |
| KLA6 | KLA6 | PUG | Uganda | Kampala | adult | Pig | ASV_1/ASV_6 | ASV_3/ASV_18 | 47 |
| KLA7 | KLA7 | PUG | Uganda | Kampala | adult | Pig | ASV_2/ASV_2 | ASV_6/ASV_14 | 58 |
| PUG3.2 | PUG3.2 | PUG | Uganda | Kabale | adult | Pig | ASV_1/ASV_2 | ASV_2/ASV_3 | 28 |
| PUG4.1 | PUG4.1 | PUG | Uganda | Kabale | adult | Pig | ASV_1/ASV_1 | ASV_2/ASV_3 | 8 |
| PUG5.3 | PUG5.3 | PUG | Uganda | Kabale | adult | Pig | ASV_1/ASV_1 | ASV_2/ASV_2 | 7 |
| PUG6.1 | PUG6.1 | PUG | Uganda | Kabale | adult | Pig | ASV_1/ASV_1 | ASV_2/ASV_2 | 7 |
| Farm A10 | Farm A10 | Farm A | United Kingdom | Devon | larvae | Pig | ASV_1/ASV_1 | ASV_2/ASV_2 | 7 |
| Farm A11 | Farm A11 | Farm A | United Kingdom | Devon | larvae | Pig | ASV_1/ASV_1 | ASV_1/ASV_3 | 3 |
| Farm A12 | Farm A12 | Farm A | United Kingdom | Devon | Larvae | Pig | ASV_1/ASV_3 | NA | NA |
| Farm A13 | Farm A13 | Farm A | United Kingdom | Devon | Larvae | Pig | ASV_1/ASV_1 | NA | NA |
| Farm A16 | Farm A16 | Farm A | United Kingdom | Devon | larvae | Pig | ASV_1/ASV_1 | ASV_3/ASV_3 | 11 |
| Farm A17 | Farm A17 | Farm A | United Kingdom | Devon | Larvae | Pig | ASV_1/ASV_1 | NA | NA |
| Farm A23 | Farm A23 | Farm A | United Kingdom | Devon | larvae | Pig | ASV_1/ASV_1 | ASV_2/ASV_3 | 8 |
| Farm A25 | Farm A25 | Farm A | United Kingdom | Devon | larvae | Pig | ASV_1/ASV_1 | ASV_3/ASV_3 | 11 |
| Farm A27 | Farm A27 | Farm A | United Kingdom | Devon | larvae | Pig | ASV_1/ASV_1 | ASV_2/ASV_3 | 8 |
| Farm A29 | Farm A29 | Farm A | United Kingdom | Devon | larvae | Pig | ASV_3/ASV_3 | ASV_1/ASV_3 | 60 |
| Farm A30 | Farm A30 | Farm A | United Kingdom | Devon | larvae | Pig | ASV_1/ASV_1 | ASV_3/ASV_3 | 11 |
| Farm A31 | Farm A31 | Farm A | United Kingdom | Devon | larvae | Pig | ASV_1/ASV_6 | ASV_2/ASV_3 | 46 |
| Farm A33 | Farm A33 | Farm A | United Kingdom | Devon | larvae | Pig | ASV_1/ASV_1 | ASV_1/ASV_3 | 3 |
| Farm A35 | Farm A35 | Farm A | United Kingdom | Devon | larvae | Pig | ASV_1/ASV_1 | ASV_1/ASV_3 | 3 |
| Farm A38 | Farm A38 | Farm A | United Kingdom | Devon | larvae | Pig | ASV_1/ASV_1 | ASV_1/ASV_3 | 3 |
| Farm A40 | Farm A40 | Farm A | United Kingdom | Devon | larvae | Pig | ASV_1/ASV_1 | ASV_3/ASV_3 | 11 |
| Farm A44 | Farm A44 | Farm A | United Kingdom | Devon | larvae | Pig | ASV_1/ASV_1 | ASV_3/ASV_3 | 11 |
| Farm A45 | Farm A45 | Farm A | United Kingdom | Devon | Larvae | Pig | ASV_1/ASV_2 | NA | NA |
| Farm A6 | Farm A6 | Farm A | United Kingdom | Devon | larvae | Pig | ASV_1/ASV_1 | ASV_2/ASV_3 | 8 |
| Farm A7 | Farm A7 | Farm A | United Kingdom | Devon | larvae | Pig | ASV_1/ASV_1 | ASV_3/ASV_3 | 11 |
| Farm B10 | Farm B10 | Farm B | United Kingdom | Derbyshire | larvae | Pig | ASV_1/ASV_2 | ASV_2/ASV_2 | 27 |
| Farm B12 | Farm B12 | Farm B | United Kingdom | Derbyshire | larvae | Pig | ASV_1/ASV_1 | ASV_2/ASV_2 | 7 |
| Farm B14 | Farm B14 | Farm B | United Kingdom | Derbyshire | larvae | Pig | ASV_1/ASV_2 | ASV_2/ASV_2 | 27 |
| Farm B16 | Farm B16 | Farm B | United Kingdom | Derbyshire | larvae | Pig | ASV_1/ASV_1 | ASV_2/ASV_2 | 7 |
| Farm B17 | Farm B17 | Farm B | United Kingdom | Derbyshire | larvae | Pig | ASV_1/ASV_2 | ASV_2/ASV_2 | 27 |
| Farm B18 | Farm B18 | Farm B | United Kingdom | Derbyshire | Larvae | Pig | ASV_2/ASV_2 | NA | NA |
| Farm B19 | Farm B19 | Farm B | United Kingdom | Derbyshire | larvae | Pig | ASV_1/ASV_2 | ASV_2/ASV_2 | 27 |
| Farm B20 | Farm B20 | Farm B | United Kingdom | Derbyshire | larvae | Pig | ASV_1/ASV_2 | ASV_2/ASV_2 | 27 |
| Farm B22 | Farm B22 | Farm B | United Kingdom | Derbyshire | larvae | Pig | ASV_1/ASV_2 | ASV_2/ASV_2 | 27 |
| Farm B4 | Farm B4 | Farm B | United Kingdom | Derbyshire | larvae | Pig | ASV_1/ASV_2 | ASV_2/ASV_2 | 27 |
| Farm B50 | Farm B50 | Farm B | United Kingdom | Derbyshire | larvae | Pig | ASV_1/ASV_2 | ASV_2/ASV_2 | 27 |
| Farm B52 | Farm B52 | Farm B | United Kingdom | Derbyshire | larvae | Pig | ASV_1/ASV_2 | ASV_2/ASV_3 | 28 |
| Farm B53 | Farm B53 | Farm B | United Kingdom | Derbyshire | larvae | Pig | ASV_1/ASV_2 | ASV_2/ASV_2 | 27 |
| Farm B54 | Farm B54 | Farm B | United Kingdom | Derbyshire | larvae | Pig | ASV_1/ASV_2 | ASV_2/ASV_2 | 27 |
| Farm B55 | Farm B55 | Farm B | United Kingdom | Derbyshire | larvae | Pig | ASV_1/ASV_2 | ASV_2/ASV_2 | 27 |
| Farm B56 | Farm B56 | Farm B | United Kingdom | Derbyshire | larvae | Pig | ASV_1/ASV_2 | ASV_2/ASV_2 | 27 |
| Farm B57 | Farm B57 | Farm B | United Kingdom | Derbyshire | larvae | Pig | ASV_1/ASV_2 | ASV_2/ASV_2 | 27 |
| Farm B58 | Farm B58 | Farm B | United Kingdom | Derbyshire | larvae | Pig | ASV_1/ASV_2 | ASV_2/ASV_2 | 27 |
| Farm B59 | Farm B59 | Farm B | United Kingdom | Derbyshire | Larvae | Pig | NA | ASV_2/ASV_2 | NA |
| Farm B64 | Farm B64 | Farm B | United Kingdom | Derbyshire | larvae | Pig | ASV_1/ASV_2 | ASV_2/ASV_2 | 27 |
| Farm C1 | Farm C1 | Farm C | United Kingdom | Clwyd | larvae | Pig | ASV_3/ASV_3 | ASV_2/ASV_2 | 61 |
| Farm C10 | Farm C10 | Farm C | United Kingdom | Clwyd | Larvae | Pig | ASV_3/ASV_3 | NA | NA |
| Farm C17 | Farm C17 | Farm C | United Kingdom | Clwyd | Larvae | Pig | NA | ASV_2/ASV_2 | NA |
| Farm C18 | Farm C18 | Farm C | United Kingdom | Clwyd | larvae | Pig | ASV_3/ASV_3 | ASV_2/ASV_2 | 61 |
| Farm C19 | Farm C19 | Farm C | United Kingdom | Clwyd | larvae | Pig | ASV_3/ASV_3 | ASV_7/ASV_7 | 65 |
| Farm C2 | Farm C2 | Farm C | United Kingdom | Clwyd | larvae | Pig | ASV_3/ASV_3 | ASV_2/ASV_16 | 62 |
| Farm C20 | Farm C20 | Farm C | United Kingdom | Clwyd | larvae | Pig | ASV_3/ASV_8 | ASV_7/ASV_7 | 67 |
| Farm C21 | Farm C21 | Farm C | United Kingdom | Clwyd | larvae | Pig | ASV_3/ASV_3 | ASV_7/ASV_7 | 65 |
| Farm C22 | Farm C22 | Farm C | United Kingdom | Clwyd | larvae | Pig | ASV_3/ASV_3 | ASV_2/ASV_2 | 61 |
| Farm C23 | Farm C23 | Farm C | United Kingdom | Clwyd | larvae | Pig | ASV_1/ASV_3 | ASV_1/ASV_1 | 40 |
| Farm C24 | Farm C24 | Farm C | United Kingdom | Clwyd | larvae | Pig | ASV_1/ASV_3 | ASV_5/ASV_5 | 44 |
| Farm C26 | Farm C26 | Farm C | United Kingdom | Clwyd | larvae | Pig | ASV_3/ASV_3 | ASV_2/ASV_2 | 61 |
| Farm C27 | Farm C27 | Farm C | United Kingdom | Clwyd | larvae | Pig | ASV_3/ASV_3 | ASV_1/ASV_2 | 59 |
| Farm C3 | Farm C3 | Farm C | United Kingdom | Clwyd | larvae | Pig | ASV_1/ASV_3 | ASV_1/ASV_2 | 41 |
| Farm C4 | Farm C4 | Farm C | United Kingdom | Clwyd | larvae | Pig | ASV_1/ASV_3 | ASV_7/ASV_7 | 45 |
| Farm C5 | Farm C5 | Farm C | United Kingdom | Clwyd | larvae | Pig | ASV_3/ASV_3 | ASV_5/ASV_7 | 64 |
| Farm C6 | Farm C6 | Farm C | United Kingdom | Clwyd | larvae | Pig | ASV_3/ASV_8 | ASV_2/ASV_5 | 66 |
| Farm C7 | Farm C7 | Farm C | United Kingdom | Clwyd | larvae | Pig | ASV_1/ASV_3 | ASV_2/ASV_2 | 42 |
| Farm C8 | Farm C8 | Farm C | United Kingdom | Clwyd | larvae | Pig | ASV_3/ASV_3 | ASV_2/ASV_2 | 61 |
| Farm C9 | Farm C9 | Farm C | United Kingdom | Clwyd | Larvae | Pig | ASV_3/ASV_8 | NA | NA |
| Farm D1 | Farm D1 | Farm D | United Kingdom | Lincolnshire | Larvae | Pig | ASV_1/ASV_2 | NA | NA |
| Farm D10 | Farm D10 | Farm D | United Kingdom | Lincolnshire | larvae | Pig | ASV_1/ASV_1 | ASV_3/ASV_7 | 12 |
| Farm D11 | Farm D11 | Farm D | United Kingdom | Lincolnshire | larvae | Pig | ASV_1/ASV_1 | ASV_2/ASV_2 | 7 |
| Farm D12 | Farm D12 | Farm D | United Kingdom | Lincolnshire | larvae | Pig | ASV_1/ASV_1 | ASV_2/ASV_2 | 7 |
| Farm D13 | Farm D13 | Farm D | United Kingdom | Lincolnshire | larvae | Pig | ASV_3/ASV_3 | ASV_3/ASV_3 | 63 |
| Farm D14 | Farm D14 | Farm D | United Kingdom | Lincolnshire | Larvae | Pig | ASV_1/ASV_1 | NA | NA |
| Farm D15 | Farm D15 | Farm D | United Kingdom | Lincolnshire | Larvae | Pig | ASV_2/ASV_2 | NA | NA |
| Farm D18 | Farm D18 | Farm D | United Kingdom | Lincolnshire | larvae | Pig | ASV_1/ASV_1 | ASV_6/ASV_7 | 16 |
| Farm D19 | Farm D19 | Farm D | United Kingdom | Lincolnshire | larvae | Pig | ASV_1/ASV_1 | ASV_7/ASV_7 | 17 |
| Farm D2 | Farm D2 | Farm D | United Kingdom | Lincolnshire | Larvae | Pig | NA | ASV_2/ASV_2 | NA |
| Farm D22 | Farm D22 | Farm D | United Kingdom | Lincolnshire | larvae | Pig | ASV_1/ASV_1 | ASV_4/ASV_4 | 13 |
| Farm D24 | Farm D24 | Farm D | United Kingdom | Lincolnshire | larvae | Pig | ASV_1/ASV_3 | ASV_2/ASV_6 | 43 |
| Farm D25 | Farm D25 | Farm D | United Kingdom | Lincolnshire | larvae | Pig | ASV_1/ASV_2 | ASV_3/ASV_7 | 33 |
| Farm D3 | Farm D3 | Farm D | United Kingdom | Lincolnshire | larvae | Pig | ASV_1/ASV_6 | ASV_7/ASV_7 | 48 |
| Farm D30 | Farm D30 | Farm D | United Kingdom | Lincolnshire | larvae | Pig | ASV_1/ASV_1 | ASV_2/ASV_2 | 7 |
| Farm D31 | Farm D31 | Farm D | United Kingdom | Lincolnshire | larvae | Pig | ASV_1/ASV_2 | ASV_7/ASV_7 | 39 |
| Farm D4 | Farm D4 | Farm D | United Kingdom | Lincolnshire | Larvae | Pig | ASV_1/ASV_1 | NA | NA |
| Farm D5 | Farm D5 | Farm D | United Kingdom | Lincolnshire | larvae | Pig | ASV_1/ASV_1 | ASV_2/ASV_2 | 7 |
| Farm D6 | Farm D6 | Farm D | United Kingdom | Lincolnshire | larvae | Pig | ASV_1/ASV_1 | ASV_3/ASV_7 | 12 |
| HUK10.1 | HUK10.1 | HUK | United Kingdom | Cornwall | Adult | Human | ASV_1/ASV_2 | NA | NA |
| HUK11.1 | HUK11.1 | HUK | United Kingdom | Cornwall | Adult | Human | ASV_1/ASV_1 | NA | NA |
| HUK4.1 | HUK4.1 | HUK | United Kingdom | Cornwall | adult | Human | ASV_1/ASV_1 | ASV_2/ASV_2 | 7 |
| HUK7.1 | HUK7.1 | HUK | United Kingdom | Cornwall | adult | Human | ASV_1/ASV_1 | ASV_2/ASV_3 | 8 |
| HUK8.1 | HUK8.1 | HUK | United Kingdom | Cornwall | adult | Human | ASV_1/ASV_1 | ASV_2/ASV_3 | 8 |
| PUK1.1 | PUK1.1 | PUK | United Kingdom | Bedfordshire | adult | Pig | ASV_2/ASV_2 | ASV_1/ASV_1 | 50 |
| PUK1.2T | PUK1.2T | PUK | United Kingdom | Bedfordshire | adult | Pig | ASV_1/ASV_1 | ASV_1/ASV_5 | 5 |
| PUK1.3 | PUK1.3 | PUK | United Kingdom | Bedfordshire | adult | Pig | ASV_1/ASV_1 | ASV_1/ASV_1 | 1 |
| PUK1.4 | PUK1.4 | PUK | United Kingdom | Bedfordshire | adult | Pig | ASV_1/ASV_1 | ASV_1/ASV_1 | 1 |
| PUK2.5 | PUK2.5 | PUK | United Kingdom | Bedfordshire | adult | Pig | ASV_1/ASV_2 | ASV_1/ASV_11 | 26 |
| PUK2.6 | PUK2.6 | PUK | United Kingdom | Bedfordshire | adult | Pig | ASV_1/ASV_1 | ASV_1/ASV_1 | 1 |
| PUK2.7 | PUK2.7 | PUK | United Kingdom | Bedfordshire | adult | Pig | ASV_1/ASV_2 | ASV_1/ASV_6 | 24 |
| PUK2.8 | PUK2.8 | PUK | United Kingdom | Bedfordshire | adult | Pig | ASV_1/ASV_2 | ASV_1/ASV_1 | 21 |
| PUK2.9 | PUK2.9 | PUK | United Kingdom | Bedfordshire | adult | Pig | ASV_1/ASV_1 | ASV_1/ASV_1 | 1 |
| PUK3.2 | PUK3.2 | PUK | United Kingdom | Bedfordshire | adult | Pig | ASV_1/ASV_2 | ASV_1/ASV_5 | 23 |
| PUK3.3 | PUK3.3 | PUK | United Kingdom | Bedfordshire | adult | Pig | ASV_2/ASV_2 | ASV_1/ASV_6 | 52 |
| PUK3.4 | PUK3.4 | PUK | United Kingdom | Bedfordshire | adult | Pig | ASV_1/ASV_1 | ASV_1/ASV_1 | 1 |
| PUK4.13 | PUK4.13 | PUK | United Kingdom | Bedfordshire | adult | Pig | ASV_1/ASV_1 | ASV_5/ASV_6 | 15 |
| PUK4.15 | PUK4.15 | PUK | United Kingdom | Bedfordshire | adult | Pig | ASV_1/ASV_2 | ASV_5/ASV_5 | 35 |
| PUK4.2 | PUK4.2 | PUK | United Kingdom | Bedfordshire | adult | Pig | ASV_4/ASV_4 | ASV_1/ASV_1 | 68 |
| PUK4.22 | PUK4.22 | PUK | United Kingdom | Bedfordshire | adult | Pig | ASV_1/ASV_1 | ASV_5/ASV_6 | 15 |
| PUK4.23 | PUK4.23 | PUK | United Kingdom | Bedfordshire | adult | Pig | ASV_1/ASV_1 | ASV_2/ASV_5 | 9 |
| PUK4.3 | PUK4.3 | PUK | United Kingdom | Bedfordshire | adult | Pig | ASV_1/ASV_1 | ASV_4/ASV_4 | 13 |
| PUK4.4 | PUK4.4 | PUK | United Kingdom | Bedfordshire | adult | Pig | ASV_1/ASV_1 | ASV_4/ASV_5 | 14 |
| PUK4.5 | PUK4.5 | PUK | United Kingdom | Bedfordshire | adult | Pig | ASV_1/ASV_1 | ASV_1/ASV_5 | 5 |
| PUK4.6 | PUK4.6 | PUK | United Kingdom | Bedfordshire | adult | Pig | ASV_2/ASV_2 | ASV_1/ASV_1 | 50 |
| PUK4.8 | PUK4.8 | PUK | United Kingdom | Bedfordshire | adult | Pig | ASV_1/ASV_2 | ASV_1/ASV_1 | 21 |
| PUK4.9 | PUK4.9 | PUK | United Kingdom | Bedfordshire | adult | Pig | ASV_1/ASV_7 | ASV_1/ASV_2 | 49 |

* denotes data taken from Roose *et al*. (Roose *et al.*, 2021). Note that all ASV numbers relate to those found in this study and therefore ASV numbers from Roose *et al*. (Roose *et al.*, 2021) had to be modified to provide uniform data.


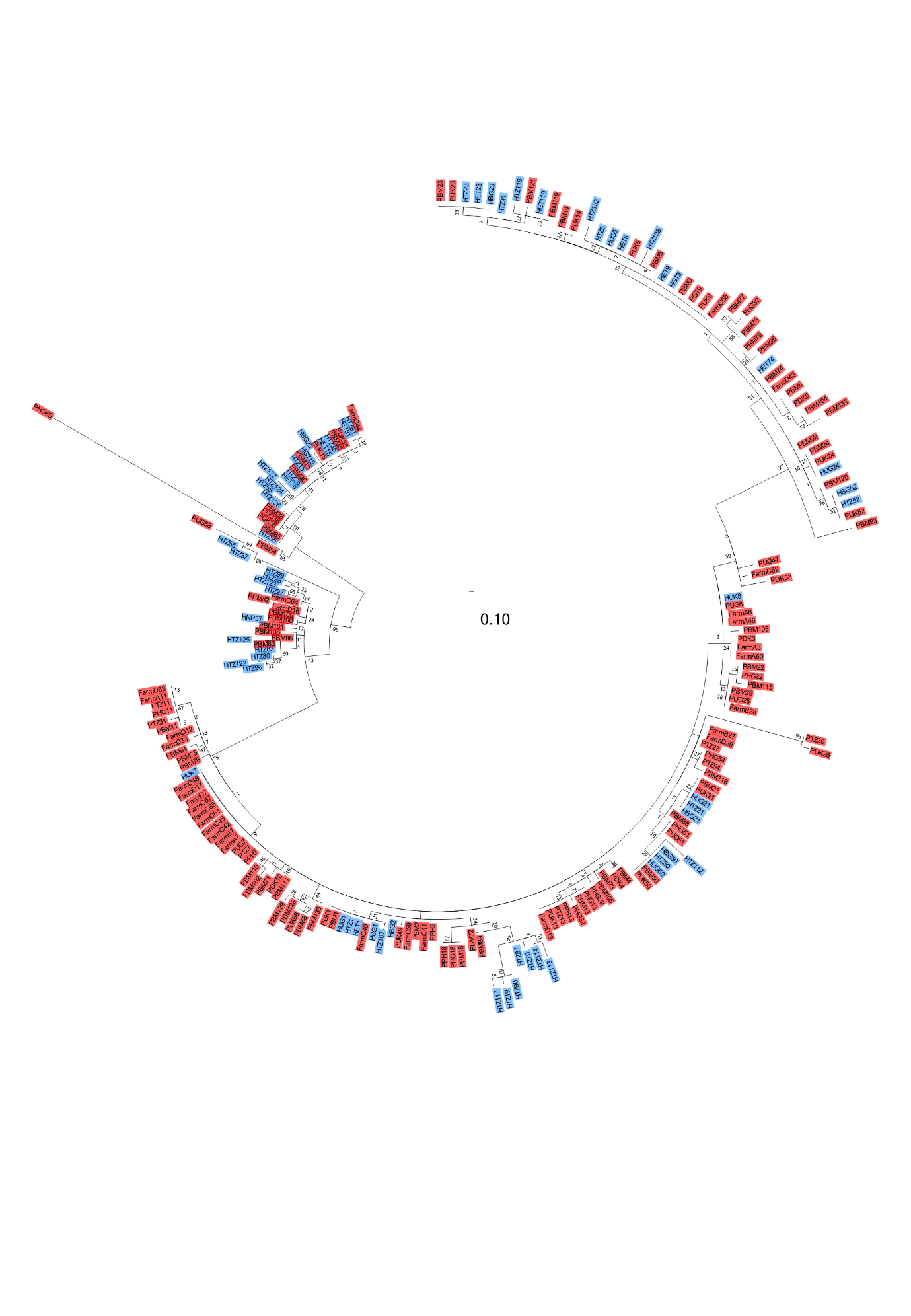


**Fig. S3** Maximum likelihood phylogeny of *Ascaris* β-tubulin genotypes. The T92+G was found to be the best fitting evolutionary model and was used to construct the maximum likelihood phylogeny with 1000 bootstraps. Abbreviations: H – humans, P – pig, BG – Bangladesh, BM – Belgium, DK – Denmark, ET – Ethiopia, GT – Guatemala, HG – Hungary, NP – Nepal, PH – Philippines, TZ – Tanzania, UG – Uganda, UK – United Kingdom. Farm A, B, C and D indicate the separate pig farms from the UK. Numbers refer to genotype number. Human genotypes are highlighted in blue and pig genotypes are highlighted in red.
